# A New Homotetramer Hemoglobin in the Pulmonary Surfactant of Plateau Zokors (*Myospalax Baileyi*)

**DOI:** 10.3389/fgene.2022.824049

**Published:** 2022-03-15

**Authors:** Jimei Li, Zhifang An, Linna Wei, Bo Xu, Zhijie Wang, Conghui Gao, Lian Wei, Delin Qi, Peng Shi, Tongzuo Zhang, Dengbang Wei

**Affiliations:** ^1^ State Key Laboratory of Plateau Ecology and Agriculture, Qinghai University, Xining, China; ^2^ Research Center for High Altitude Medicine, Qinghai University, Xining, China; ^3^ Key Laboratory of Adaptation and Evolution of Plateau Biota, Northwest Institute of Plateau Biology, Chinese Academy of Sciences, Xining, China; ^4^ State Key Laboratory of Genetic Resources and Evolution, Kunming Institute of Zoology, Chinese Academy of Sciences, Kunming, China

**Keywords:** plateau zokor (*Myospalax Baileyi*), homotetramer hemoglobin, *γ-like* gene, alveolar epithelial type II (ATII), osmiophilic multilamellar body (LBs), pulmonary surfactant (PS), hypoxic adaptation

## Abstract

The plateau zokor (*Myospalax baileyi*) is a native species to the Qinghai-Tibetan Plateau, inhabiting hypoxia and hypercapnia sealed subterranean burrows that pose several unique physiological challenges. In this study, we observed a novel heme-containing protein in the pulmonary surfactant (PS) of plateau zokor, identified the encoding gene of the protein, predicted its origination and structure, verified its expression in alveolar epithelial cells, and determined the protein’s affinity to oxygen and its effect on the oxygen-dissolving capability in the PS of plateau zokors. The protein is an unusual homotetramer hemoglobin consisting of four *γ-like* subunits, and the subunit is encoded by a paralog gene of *γ,* that is *γ-like*. The divergence time of *γ-like* from *γ* is estimated by the molecular clock to be about 2.45 Mya. The generation of *γ-like* in plateau zokors might well relate to long-time stress of the high land hypoxia. Unlike *γ,* the *γ-like* has a hypoxia response element (HRE) and a lung tissue-specific enhancer in its upstream region, and it is expressed specifically in lung tissues and up-regulated by hypoxia. The protein is named as γ_4_-like which is expressed specifically in Alveolar epithelial type II (ATII) cells and secreted into the alveolar cavities through the osmiophilic multilamellar body (LBs). The γ_4_-like has a higher affinity to oxygen, and that increases significantly oxygen-dissolving capability in the PS of plateau zokors by its oxygenation function, which might be beneficial for the plateau zokors to obtain oxygen from the severe hypoxia environments by facilitating oxygen diffusion from alveoli to blood.

## Introduction

The lung is both a gas exchange and a hematopoietic organ (Lefrançais et al., 2017). Alveolar epithelium of the lung is primarily composed of Alveolar epithelial type I (ATI) cells and Alveolar epithelial type II (ATII) cells (Crandall and Matthay, 2001). The ATI cells are large squamous cells while ATII cells are cuboidal in shape (Fehrenbach, 2001). The ATI have mainly gas exchange function, and the ATII have important pulmonary surfactant (PS) secretory capacities (Fehrenbach, 2001; Warburton and Bellusci, 2004). PS is synthesized by ATII pneumocytes and secreted into the alveolar cavities through the osmiophilic multilamellar body (LBs). PS is composed of around 80% phospholipids, 5%–10% neutral lipids—mainly cholesterol, and 8%–10% proteins, with 5%–6% of total surfactant mass being constituted by specific surfactant proteins (Goerke, 1998). The protein moiety of surfactant comprises four specific surfactant-associated proteins (Haagsman and Diemel, 2001). They can be classified in two groups, the hydrophilic surfactant proteins SP-A and SP-D, and the hydrophobic surfactant proteins SP-B and SP-C (Serrano and Pérez-Gil, 2006). The phospholipids and proteins have important functions of reducing the surface tension (Goerke, 1998), defending against pathogens (Kingma and Whitsett, 2006), maintaining the surface physical stabilization (Pérez-Gil, 2008), and facilitating respiratory gas exchange by increasing oxygen-dissolving capability and oxygen diffusion in the pulmonary surfactant (Olmeda et al., 2010). The gas exchange performance depends not only on the total alveolar surface area but also on the compositions and contents of PS (Zuo et al., 2008). Many studies have revealed that, compared to plain animals, species native to high-altitude environments as well as mole rats inhabiting deep and sealed subterranean burrows have a larger total alveolar surface area due to the increased whole lung volume and the higher density of alveoli (Widmer et al., 1997). Plateau zokor (*Myospalax baileyi*), a species endemic to the Qinghai–Tibetan Plateau, belongs to the order Rodentia and the family Cricetidae (Liu, 1995; Wang et al., 2008). It is a solitary, fossorial rodent that spends its entire lives underground in sealed hypoxic burrows at 2,800 to 4,200 m (Fan and Shi, 1982). The average oxygen concentration in the burrows of plateau zokors is found to be 20% lower than that of the local atmospheres (Zeng et al., 1984).

However, plateau zokors have significantly higher arterial oxygen partial pressure, arterial oxygen saturation, and oxygen utilization rate than plain animals (Wei et al., 2006). Over evolutionary time, the plateau zokor has developed a series of physiological and molecular mechanisms for effective oxygen absorption, transfer, and utilization (Wei et al., 2006; Qi et al., 2008; Shao et al., 2015). Recent studies showed that the genes involved in respiratory gaseous exchange are significantly positively selected in the plateau zokor; this finding was not found in previous analyses for positive selection in other plateau species, such as the yak (*Bos grunniens*), Tibetan boar (*Sus scrofa*), and Tibetan antelope (*Pantholops hodgsonii*) (Qiu et al., 2012; Ge et al., 2013; Li et al., 2013; Shao et al., 2015), suggesting that this adaptation might be specific to the plateau zokor.

We have reported that a heme-containing protein was observed in the PS of plateau zokors, and the amount of dissolved oxygen in the PS of plateau zokors was significantly higher than that in Sprague-Dawley rats and saline (Li et al., 2021). It is known to all that the heme-containing proteins like myoglobin, cytoglobin, and neuroglobin have the function of oxygen storage which keeps oxygen supply to cells. We speculate that the heme-containing protein in the PS of plateau zokors might be advantageous for getting enough oxygen from the severe hypoxia environments. However, the subunit constitutions, the encoding gene and its expression patterns, and the physiological functions of the heme-containing protein are still unclear. Therefore, in the present paper, to explore insights into the new adaptive mechanisms of the plateau zokor to hypoxia, we tested the origin, structure, and the specific expression of the gene that encodes the heme-containing protein, and the oxygen affinity of the protein as well as its effect on the amount of dissolved oxygen in the PS of plateau zokors.

## Materials and Methods

### Animal Procedures and Sampling

A number of plateau zokors (regardless of sexuality) were live-trapped at the Laji Mountain area at elevations of 2,700–3,000 m (36°26′ N, 101°69′E) and 3,600–3,800 m (36°72′ N, 101°28′E) in the Guide County, Qinghai Province, China. At the two spots, the average oxygen partial pressure and average oxygen concentration in the atmosphere are 15.25 kPa and 212.6 g/m^3^, and 13.42 kPa and 180.6 g/m^3^, respectively. The weight of the captured plateau zokors were in the 200–250 g range. Among those plateau zokors, 30 trapped at 3,600–3,800 m were used for preparing PS, six for conducting immunofluorescence, 16 (eight at each elevation) for determining the expression levels of the gene that encodes the heme-containing protein, and 18 for isolating alveolar I and II type epithelial cells (ATI and ATII) and LBs. As a control group, 44 Sprague–Dawley rats were purchased from the Experimental Animal Center of Lanzhou University (36°02′N, 103°51′E; 1,500 m a.s.l.) in Lanzhou City, Gansu Province, China. The weights of the Sprague-Dawley rats were in the 200–250 g range. Among those Sprague-Dawley rats, 30 were used for preparing PS, six for conducting immunofluorescence, and eight for determining the expression levels of the gene that encodes the heme-containing protein. The average oxygen partial pressure and average oxygen concentration in Lanzhou City are 17.71 kPa and 251.0 g/m^3^, respectively.

The plateau zokors and Sprague-Dawley rats that were used for analyzing the expression of the gene that encodes the heme-containing protein were anesthetized with chloral hydrate (10%), and the lung, liver, heart, kidney, brain, and skeletal muscle were collected immediately at spots and stored in liquid nitrogen. Two milliliters of blood from each species was collected in ethylene diamine tetraacetic acid (EDTA) anticoagulant tubes from the carotid artery. Hemoglobin extraction was performed according to Shorr et al. (1993) (Shorr et al., 1993). All procedures involved in the handling and care of animals were in accordance with the China Practice for the Care and Use of Laboratory Animals and approved by the China Zoological Society (permit number: GB/T35892-2018).

### Assay of Proteins in the Pulmonary Surfactant

For assaying proteins in PS, the lungs of 30 plateau zokors and that of 30 Sprague-Dawley rats were perfused intensively to avoid PS being contaminated by blood after animals were anesthetized with chloral hydrate (10%). Briefly, the anesthetized animal’s chest was opened to expose the heart and lung, and then the lavage instrument cannula was inserted into the pulmonary artery from the right ventricle, the left atrium was cut open, and the lung was perfused intensively with sterile saline for 20 min. The intact lung was collected and then weighed after drying their surfaces using filter paper. The gas in the alveoli was elicited by syringe, and then the PS of the alveoli was collected by washing with 2 ml of sterile saline (at 37°C) per gram of lung tissue. The PS solutions of every five animals were mixed to a pool and then centrifuged at 10,000 rpm for 10 min at 4°C. The supernatant was lyophilized at −50°C using a vacuum freeze-dryer (GLZY-0.5B Shanghai Pudong Freeze Drying Equipment Co., Ltd.). The freeze-dried powder was stored at −20°C for assaying the protein constitutions.

The polyacrylamide gel electrophoresis (PAGE) was used to demonstrate the proteins in the PS of plateau zokors and that of Sprague-Dawley rats. Because a red band was observed in the PS of plateau zokors before Coomassie brilliant blue (CBB) staining, to confirm the red band wasn’t the hemoglobin contaminated by blood, the differences of electrophoretic mobilities and subunit constitutions between the PS heme-containing protein and hemoglobin of plateau zokors were demonstrated by PAGE and sodium dodecyl sulfate PAGE (SDS-PAGE), respectively, and the red protein was recycled from gels and its amino acid sequences (AAs) were assayed by mass spectrometry (MS) in the Beijing Huada Protein Research and Development Center, respectively. The AAs of the heme-containing protein was assembled using the protein overlapping peptide method in PeptideMass (Wilkins et al., 1997) and manually adjusted in Microsoft Excel 2016. The protein bands after CBB staining were pictured using the Bio-Rad gel imaging system, and the relative content of the PS heme-containing protein was measured by the Image Pro program, the relative content (%) equal to the gray value of the heme-containing protein band/the sum of gray values of all protein bands. The molecular weight of the heme-containing protein was measured by sepharose gel chromatography, and that of its subunit was predicted by the ProtParam tool (https://web.expasy.org/protparam/) and confirmed by MS assay, respectively. The AAs of *β*-globins in mouse, rat, and spalax were downloaded from GenBank (www.ncbi.nlm.nih.gov) (Table 1). The AAs of the heme-containing subunit plateau zokors, and the β subunits of mouse (*Mus musculus*), rat (*Rattus norvegicus*), American pika (*Ochotona princeps*), and the plateau zokor itself were aligned by DNAMAN version 9.0 (http://www.lynnon.com).

**TABLE 1:** Names of 10 kinds of animals and their GenBank IDs of *β*-globin gene cluster.

Species	Shortened form	GenBank ID of *β*-globin gene cluster
*Heterocephalus glaber*	African naked mole rat	NW_004624817
*Nannospalax galili*	Upper Galilee mountains blind mole rat	NW_008335836
*Rattus norvegicus*	Rat	NC_005100
*Mus musculus*	Mouse	NC_000073
*Fukomys damarensis*	Damaraland mole rat	NW_011044170.1
*Cavia porcellus*	Guinea pig	NT_176348.1
*Microtus ochrogaster*	Prairie voles	NW_004949171.1
*Peromyscus maniculatus*	Deer mouse	EU204642.1
*Myotis lucifugus*	Little brown bat	NW_005871186
*Homo sapiens*	Human	NG_000007

### Identification of the Gene-Encoding Subunit of the Heme-Containing Protein

The AAs of the subunit of the heme-containing protein were highly homologous with that of the β-like globins. Therefore, the sequences of *β*-globin gene clusters of 10 mammalian species, *Homo sapiens*, *Myotis lucifugus*, *Microtus ochrogaster, Rattus norvegicus*, *Mus musculus*, *Nannospalax galili*, *Heterocephalus glaber*, *Fukomys damarensis*, *Cavia porcellus*, and *Peromyscus maniculatus*, were downloaded from GenBank. The sequences of the *β*-globin gene cluster of *Myospalax baileyi* was supplied by the Kunming institute of zoology, Chinese Academy of Sciences. The genomic structures of the *β*-globin gene clusters in *Homo sapiens*, *Myotis lucifugus*, *Peromyscus maniculatus*, *Rattus norvegicus*, and *Mus musculus* have been characterized using information from the database records (Collins and Weissman, 1984; Efstratiadis et al., 1980; Hoffmann et al., 2008; Opazo et al., 2008; Shehee et al., 1989). For the other six rodents (*Myospalax baileyi*, *Nannospalax galili*, *Heterocephalus glaber*, *Fukomys damarensis*, *Cavia porcellus*, and *Microtus ochrogaster*) (Table 1), the *β*-globin genes were identified in the unannotated genomic sequences using the program Genescan (Burge and Karlin, 1997), and the known exon sequences were compared to genomic contigs using the program Blast 2 version 2.7.1 (Tatusova and Madden, 1999). To analyze the polygenetic relationships among the above 11 mammalian species, the complete mitochondrial DNA (mtDNA) sequences of those species were downloaded from the GenBank (Table 2). The mtDNA phylogenetic Bayesian inference trees of the 11 species were reconstructed using MrBayes 3.2.6 (Huelsenbeck and Ronquist, 2001). Bayesian inference with Markov-chain Monte Carlo (MCMC) (Gamerman and Lopes, 2006) sampling was performed using MrBayes 3.2.6 run for one million generations. We made two simultaneous runs, sampling trees every 1,000 generations, with three heated and one cold chain to encourage swapping among the MCMC chains and to avoid the analysis remaining in local rather than global optima. jModelTest (Darriba et al., 2012) was used to select the optimal models based on the Akaike Information Criterion (AIC) (Sakamoto et al., 1986). The convergence of sampled parameters and potential autocorrelation (effective sampling size/ESS for all parameters >200) was investigated in Tracer 1.6 (http://tree.bio.ed.ac.uk/software/tracer/). Additionally, the average standard deviation of split frequencies between both runs was checked (<0.01). The Bayesian posterior probabilities were obtained from the 50% majority rule consensus of the post-burn-in trees sampled at stationarity, after removing the first 25% of trees as a “burn-in” stage.

**TABLE 2:** Names of 11 kinds of animals and their GenBank IDs of mtDNA.

Species	Shortened form	GenBank ID of mtDNA
*Myospalax baileyi*	Plateau zokor	NC_018098
*Heterocephalus glaber*	African naked mole rat	HQ689652
*Nannospalax galili*	Upper Galilee mountains blind mole rat	JN571132
*Rattus norvegicus*	Rat	KF011917
*Mus musculus*	Mouse	J01420
*Fukomys damarensis*	Damaraland mole rat	KT321364
*Cavia porcellus*	Guinea pig	NC_000884
*Microtus ochrogaster*	Prairie voles	NC_027945
*Peromyscus maniculatus*	Deer mouse	NC_039921
*Myotis lucifugus*	Little brown bat	NC_029849
*Homo sapiens*	Human	V00662

For cloning and sequencing of *β*-like globin genes of plateau zokors, total DNA was isolated using TIANamp Genomic DNA Kit (Tiangen Biotech Co., Ltd., Beijing, China) and amplified with the degenerate primers (F, 5′-AGG​TGA​ATG​TGG​ATG​AAG​TTG​GT-3′, R, 5′-GGA​AAA​GGT​GCC​CTT​GAG​GTT​GT-3′). Each single band of PCR products was purified using a PCR purification Kit (TaKaRa Agarose Gel DNA Purification Kit Ver.2.0), and the product was ligated into pMD^®^19-T vector, and the vector was transformed into the JM109 high-efficiency competent *Escherichia coli* cells by using the PCRR II TOPOR Vector Kit (Invitrogen). Recombinant bacteria were identified by blue/white screening and confirmed by PCR. Individual colony grown on LB (Luria Bertani) agar plates was used as templates in standard PCR reactions with degenerate primers to amplify plasmid inserts. The sizes of the products were assessed on 1.5% agarose gels. Plasmids containing the insert were purified using a plasmid isolation and purification Kit (Promega) and used as a template for DNA sequencing. The sequencing was carried by Sangon Biotech (Shanghai) Co., Ltd. Nucleotide sequence analysis was performed using the DNAMAN version 9.0.

The phylogenetic relationships of *β*-like globin genes of plateau zokor were analyzed by Maximum-likelihood tree using IQ-TREE (Nguyen et al., 2015). The alignments of the *β*-globin genes were conducted using MAFFT 7.0 (Katoh and Standley, 2013), and the ModelFinder program was used to estimate the parameters of a TPM3+F + G4 model of nucleotide substitution (Kalyaanamoorthy et al., 2017). Measures of bootstrap support were based on 1,000 pseudo-replicates (Danielsson et al., 2001). The phylogenetic relationships of the *γ* (*HBG*) and *γ-like* (*HBGlike*) of the 11 species were analyzed by Bayesian inference trees based on the coding sequences, 1 kb of the upstream flanking sequences, 1 kb of the downstream flanking sequences, and intron 2 sequences. The method was the same as that used for the mtDNA tree. We used the HKY + G model (upstream flanking sequence and exon1) and a GTR + I + G model (intron 2, exon2, exon3, and downstream flanking sequence). To root the Bayesian inference tree, the corresponding major adult *β* globin sequence (*HBB*) of human was used as the outgroup. More detailed information is available in the supplemental materials and methods (https://doi.org/10.6084/m9.figshare.14034557.v2).

### Prediction of the Origination and Structures of *γ* and *γ*-Like

The coding sequences of the *γ-like* (*HBGlike*) of plateau zokor and the *HBGlike* and *HBG* of the 11 mammalian species were aligned using ClustalX 1.81. The maximum-likelihood tree of these genes was conducted using the same method described above. To root the maximum-likelihood tree, the *β* globin coding sequence (*HBB*) in human was used as the outgroup. The divergence time between the *γ* and *γ*-like of plateau zokor was predicted by molecular clock using MEGA 7.0 (Kumar et al., 2016). As a calibration point, we used a range of Euarchontoglires-Laurasiatheria divergence times spanning about 85 Mya (Wildman et al., 2007).

The structures of *γ* and *γ-like* of plateau zokor were analyzed using Genescan (http://hollywood.mit.edu/GENSCAN.html). The promoters, CAT box, TATA box, and the transcriptional start sites were predicted using TSSG online software (http://linux1.softberry.com). The transcription regulatory elements were predicted using Nsite online software and GPminer (http://gpminer.mbc.nctu.edu.tw/index.php) (Solovyev et al., 2010). The transposons, simple repeats sequences, and other transcription regulatory factors were predicted using the LTR finder program and RepeatMasker web server (http://www.repeatmasker.org) (Xu and Wang, 2007). All the transcription regulatory elements were annotated by GeneCards (https://www.genecards.org/) (Liu et al., 2015) and the tissue-specific enhancer was predicted using the Enhancer Elements Locator (http://www.cs.helsinki.fi/u/kpalin/EEL/) and human Tissue-Specific Enhancer Database (TiED, http://lcbb.swjtu.edu.cn/TiED/). The gene structure was visualized by IBS and CorelDrawX8 (Bouton, 2011; Liu et al., 2015).

### Measure of *γ-like* Expression Levels in Lung Tissues

For analyzing the tissue-specific expression of *γ-like* in lung, liver, heart, kidney, brain and skeletal muscle, and its expression levels in lungs of plateau zokors at difference altitudes, the specific primers for *α, β, γ-like,* and *β-actin* were designed using the coding sequences of the plateau zokors and Sprague-Dawley rat; the sequences of the primers are shown in Table 3. The primers of *γ-like* were designed in the low-homologous regions in these *β* globin genes. The sense primers and the anti-sense primers between *γ-like* and *γ* of plateau zokor have differences of 6 bp (33.3%) and 5 bp in (26.3%), respectively. The *γ-like* could be cloned specifically by the primers, which was evaluated by the primer blast program web server in NCBI. The primers were synthesized by Shanghai Bioengineering Co., Ltd. Total RNA was isolated using TRIzol reagent (Invitrogen Corp, Waltham, MA, USA). RNA concentration and purity were assessed by UV spectrophotometry (1.8 < A260/A280 < 2.0). RNA integrity was checked using electrophoresis. Reverse transcription reaction was carried out starting from 4 μg of total RNA using the First Strand cDNA Synthesis kit (Thermo Scientific, Waltham, MA, USA). To make standard curves, 1 μl of first-strand cDNA was amplified with Premix Ex Taq Version Kit (TaKaRa Bio Inc., Shiga, Japan), and quantification of PCR products were used for plotting standard curves. The initial product concentration was set at 1 and standard curves were generated using a 10-fold serial dilution ranging from 1 to 10^–7^. Real-time PCR was performed using the Bio-Rad IQ™ 5 instrument and software (IQ5 Optical System Software; version 2.1). The PCR cycles were composed of predenaturation at 95°C for 3 min, 35 cycles of 95°C for 30 s, 58°C for 1 min, and 72°C for 30 s, followed by a final elongation at 72°C for 10 min. *β-actin* was used as an internal control. *γ-like* mRNA level was normalized with *β-actin* mRNA to compensate for variations in initial RNA amounts. Normalization was carried out by dividing the logarithmic value of *γ-like* by the logarithmic value of *β-actin.*

**TABLE 3:** The primers of *α*, *β*, *γ-like*, and *β-actin* used in the present study.

Species	Gene	Sense primer	Anti-sense primer
Zokor	*α*	5′-CCA​CCA​CCA​AGA​CCT​ACT​TCC-3′	5′-CAG​AGC​AGA​CAG​GGC​ACT-3′
	*γ-like*	5′-TTA​AGG​ACC​TGG​ACA​ACC-3′	5′-AGG​ACA​ATC​ACC​ATC​GTA​T-3′
Sprague-Dawley rat	*α*	5′-TCA​AGG​CTC​ACG​GCA​AGA-3′	5′-GGC​AGT​GGC​TCA​GGA​ACT-3′
	*β*	5′-TGT​CCT​CTG​CCT​CTG​CTA​TC-3′	5′-GCT​TGT​CAC​AGT​GGA​GTT​CA-3′
	*β-actin*	5′-ACG​GTC​AGG​TCA​TCA​CTA​TCG-3′	5′-ACT​GTG​TTG​GCA​TAG​AGG​TCT​T-3′

For analyzing the expression levels of the γ-like in lungs of plateau zokors, the polyclonal antibody against β subunits and γ-like subunit of plateau zokor and Sprague-Dawley rat were designed using their homologous segment WGKVNVDEVGGETLG. Antibody was prepared by Beijing Liuhe Huada Gene Technology Co., Ltd. The polypeptide was synthesized and coupled with keyhole limpet hemocyanin (KLH) for immunization. Then, the KLH-coupling polypeptide was injected into a male New Zealand white rabbit four times in 40 days to prepare the polyclonal antibodies. The purity and titer of the polyclonal antibody was detected by SDS-PAGE and enzyme-linked immunosorbent assay (ELISA) technology, respectively. The specificity of the polyclonal antibody against the *γ-like* subunit of plateau zokor was identified by using a capillary-based automated system (Simon, ProteinSimple) and Compass for SW software (http://www.proteinsimple.com/simon.html). The results showed the purity of the antibody was higher than 90% and the titer of the antibody was 1: 51200. The single band observed on the entire lanes with molecular weight marker indicated that the polyclonal antibody against the *γ-like* subunit of plateau zokor has a high specificity (Supplementary Figure S1). Therefore, the polyclonal antibody was applicable for Western blot and immunofluorescence analysis.

For analyzing the expression levels of γ-like by immunofluorescence, the lungs of six plateau zokors and those of the same number Sprague-Dawley rats were perfused intensively with sterile saline *via* the right ventricle to wash the blood, and then the lungs were irrigated with 4% PFA for fixation. The tissues were dehydrated to 100% ethanol *via* a series concentration of ethanol (30%, 50%, 70%, 95%, 100%, and 100%), cleared in xylene and then embedded in paraffin wax after immersion in the paraffin for 12 h. Microtome sections (5 µm) were obtained and mounted on glass slides. The slides were stained with hematoxylin and eosin. Briefly, the 5-μm slices were deparaffinized in xylene and rehydrated *via* a series of concentrations of ethanol (100%, 100%, 95%, 70%, 50%, and 30%) to ddH_2_O. The slides were deparaffinized in xylene and then rehydrated for 5 min each in a graded series of ethyl alcohol with a final wash in ddH_2_O. Antigen retrieval was accomplished by incubating slides in 0.01 M sodium citrate and heating in a microwave oven on high for 20 min. The slides were cooled in sodium citrate solution for 20 min, washed in phosphate-buffered saline (PBS: NaH_2_PO_4_, Na_2_HPO_4_, NaCl; pH 7.4), and then incubated in 3% hydrogen peroxide for 10 min, before being washed in PBS. The sections were washed three times with PBS and incubated with 10% goat blocking serum for 2 h at room temperature. Then, the sections were incubated with β-like antibody (diluted 1: 600) and normal rabbit IgG (1:500; Santa Cruz Biotechnology, Sta. Cruz, CA, USA) overnight at 4°C. After washing in PBS, the sections were incubated with the secondary antibodies (donkey anti-Rabbit IgG, 1:500; Proteintech, Chicago, IL, USA) for 2 h at room temperature in the dark. Sections were washed thrice with PBS and incubated with DAPI (Bioss, China), mounted with an antifade solution (Boster, China), and photographed with a fluorescence microscope (Nikon E 200; Nikon, Tokyo, Japan). The integrated optical density (IOD) per unit pixel was measured using the Image-Pro Plus 6.0 image analysis system (IPP 6.0; Media Cybernetics Inc., Rockville, MD, USA).

The relative expression levels of γ-like subunit in lung tissues of plateau zokors were measured by automated capillary Western blot system, WES System (Protein Simple), which utilizes capillary based electrophoretic separation and detection of proteins. The simple Western immunoblots were performed on a PeggySue (Protein Simple) using the Size Separation Master Kit with Split Buffer (12–230 kDa) according to the manufacturer’s standard instruction. The lung tissue extracts with equal protein concentration were mixed with 0.1×sample buffer and 5×fluorescentmaster mix. The protein samples and the biotinylated ladder were denatured by heating at 95°C for 5 min. Protein samples, biotinylated ladder, γ-like antibody (1: 50), horseradish peroxidase (HRP)-conjugated secondary antibodies, chemiluminescence substrate, and wash buffer were dispensed into respective wells of the assay plate and placed in Wes equipment. Signal intensity (area) of the protein was normalized to the peak area of loading control of *β*-actin (1:50, Abcam, UK). Quantitative analysis was performed using the Compass software (Protein Simple).

### Verification of *γ-Like* Expression in ATI, ATII, and LBs

For isolating the alveolar epithelial cells, the lung tissues of plateau zokors were perfused intensively with solution I (NaCl 137, KCl 2.7, Na_2_HPO_4_ 8.1, KH_2_PO_4_ 1.56 mM, Heparin sodium 12,500 U/ml, pH 7.40). During perfusion, the lungs were inflated with air from the tracheal cannula to total lung capacity (4–5 ml) several times to perfuse the lungs completely and remove the blood. The intact lung was moved carefully and washed in PBS three times. Next, the lung was washed with solution II (solution I plus EDTA_2_Na 0.2 mM and EGTA 0.2 mM). A total of 12–15 ml of 0.25% trypsin-EDTA (Gibco, Grand Island, NY, USA) was injected continually *via* the trachea for 20 min at 37°C for three times. The trachea and large airways were then discarded; each lung was minced with sharp scissors to a final size of 1 mm^3^ in Dulbecco’s Modified Eagles Medium (DMEM) (Gibco) without fetal bovine serum (FBS) (Gibco). Two milliliters of FBS were added to stop the trypsin reaction, and the minced tissue suspension was moved into a 50-ml centrifuge tube and shaken in a reciprocating water bath (37°C) at 130 cycles per minute for 5 min. The lung minces and cell suspension were filtered sequentially through 100-, 70-, and 40-μm filters, and centrifuged at 1,100 rpm for 8 min. The supernatant was discarded, and the pelleted cells were then resuspended in Dulbecco’s phosphate-buffered saline (DPBS) (Gibco). A volume of twice as much ACK Lysis Buffer (Solarbio, China) was added; the tube was incubated at room temperature for 5 min and then centrifuged at 1,100 rpm for 5 min. The cells were incubated in DMEM (with 20% FBS) in a 25 cm^2^ culture flask at 37°C, 45 min for three times. The unattached cells were incubated in a rat IgG-coated polystyrene bacteriological 100 mm petri dish (0.5 mg/ml rat IgG/dish) at 37°C for 2 h. The unattached cells were collected and centrifuged at 1,100 rpm for 8 min, and the supernatant was discarded. Finally, the alveolar epithelial cells were divided into three parts: one part of alveolar epithelial cells was used for ATI sorting, another part was used for ATII sorting, and the last one was used for isolating LBs.

For isolating highly pure primary ATI and ATII by the Flow Cytometric Cell Sorting (BD FACSAria: BD Biosciences, San Jose, CA, USA), the aquaporin 5 (AQP-5) is used as the specific markers of ATI, and the surfactant protein C (SP-C) are used as that of ATII, respectively. The isolated alveolar epithelial cells were resuspended in 2 ml AQP-5 (Proteintech) or 2 ml SP-C (Proteintech) antibody cocktail for flow cytometric cell-sorting, respectively, and the cells were incubated at 37°C for 1 h in the dark. Wash the cells by filling the 15 ml centrifuge tube with DMEM medium and centrifugation for 10 min at 1,100 rpm and 4°C for three times. Remove the supernatant and resuspend the cells in 2 ml secondary antibody cocktail (donkey anti-Rabbit IgG corallite 488-conjugate, Proteintech). Stain for 1 h at 4°C in the dark. Wash the cells through filling the centrifuge tube with DMEM and centrifugation for 10 min at 1,100 rpm and 4°C for three times. Resuspend the cells in 2 ml of DPBS and prefilter through a 70 μm filter into a FACS tube for cell-sorting to avoid any cell clumps that might clog the cell sorter. Select a 100 μm nozzle for cell-sorting of ATI and ATII. Set up compensation using green color controls. Gate on the SSC cells that are negative for the fluorochromes used for staining and sort into a tube containing DMEM. Use forward scatter height (FSC-H) *vs*. area (FSC-A) and sideward scatter height (SSC-H) *vs*. area (SSC-A) windows to exclude any doublets or cell aggregates. The sorted cells were collected into a FACS tube containing 500 μl ice-cold complete DMEM.

For testing the purity of the ATI and ATII cells by immunocytochemistry, the isolated cell samples were fixed in 4% PFA for 30 min at room temperature, and the cells were washed with PBS 5 min for three times. Then the samples were permeabilized in 0.5% Triton X-100 for 20 min, blocked in 10% goat blocking serum for 1 h at room temperature, and washed with PBS 5 min for three times. The ATI samples were incubated with SP-C antibody (1:50), and the ATII samples were incubated with AQP-5 antibody (1:100) at 4°C overnight. After three washes in PBS, samples were incubated with the secondary antibody (donkey anti-Rabbit IgG corallite 594-conjugate, 1:500; Proteintech) at 37°C for 1.5 h in the dark. Finally, samples were incubated with DAPI, mounted with an antifade solution (Boster, China), and photographed with fluorescence microscope (Leica Microsystems, Germany). The purities of ATI and ATII cells were calculated by the number of cells showing double-labeling fluorescences (green and red) divided by the total cells.

For isolating and purifying of LBs, the isolated alveolar epithelial cells were placed on ice and washed twice with DPBS and once with 10 mM N-(2-hydroxyethyl) piperazine-*N*′-(2-ethanesulfonic acid) (HEPES) (pH 7.4), and homogenized with a dounce glass homogenizer (10 strokes with pestle A, 100 strokes with pestle B). The homogenate was centrifuged for 10 min at 4,000 rpm and 4°C for three times. Then, the homogenate was incubated with SP-C antibody and stained with donkey anti-Rabbit IgG corallite 488-conjugate. The Flow Cytometric Cell Sorting (BD FACSAria) was used to purify of LBs, and the protocol of the FACS sorting was performed according to the methods described in ATI and ATII cells sorting.

The purified ATI, ATII, and LBs were identified by transmission electron microscopy (TEM), respectively. Firstly, the ATI, ATII, and LB samples were fixed in 2.5% glutaraldehyde. The subsequent process was according to Chen et al. (2004) (Chen et al., 2004), including postfixation with 1% osmium tetroxide for 1 h. Then specimens were dehydrated in 30%, 50%, 70%, and 90% acetone for 10 min and dehydrated in 100% acetone for three times. The specimens were embedded in Epon-Araldite resin at 37°C for 12 h and at 60°C for 48 h. Ultra-thin (80 nm) sections were cut (Leica, EM UC7, Heidelberg, Germany) and dyed with 2% uranyl acetate and 1% lead citrate. The specimens were identified using transmission electron microscope (Hitachi, H-7650, Japan).

For verifying the expression of γ-like in ATI, ATII, and LBs by immunofluorescence, 5 μl of the ATI, ATII, and LB solution samples which were isolated by flow cytometry were dropped onto the adhesive slides to air dry, respectively, fixed in 4% PFA for 30 min at room temperature, and washed with PBS 5 min for three times. The samples were then permeabilized in 0.5% Triton X-100 for 20 min, blocked in 10% goat blocking serum for 1 h at room temperature, and washed with PBS 5 min for three times. All the ATI, ATII, and LBs samples were incubated with γ-like antibody (1:100) at 4°C overnight. After three washes in PBS, samples were incubated with the secondary antibody (donkey anti-Rabbit IgG coralite 594-conjugate, 1:500; Proteintech) at 37°C for 1.5 h in the dark. Finally, samples were incubated with DAPI, mounted with an antifade solution (Boster, China), and photographed with fluorescence microscope (Leica Microsystems, Germany).

### Determination of Oxygen Dissociation Curves

The hemoglobin (Hb) and γ_4_-like of plateau zokor were purified and lyophilized using sepharose gel chromatography and a vacuum freeze-dryer, respectively. The Hb and γ_4_-like freeze-dried powder were then dissolved in deionized water and their concentrations were adjusted to 0.3 mmol/L. The 0.3 mmol/L Hb and γ_4_-like solutions were freshly prepared in 0.1 mol/L HEPES buffer (pH 7.4) in the presence of anionic cofactors Cl (0.1 mol/L KCl) and/or DPG (twofold molar excess over tetrameric Hbs) added at physiological levels and approximating conditions existing within red blood cells (Imai, 1982; Mairbäurl and Weber, 2011). The oxygen dissociation curves were measured on 2–4 μl prepared Hb and γ_4_-like solutions using a modified diffusion chamber method as previously described (Weber, 1981; Weber, 1992). A TU-1901 double-beam UV/VIS spectrophotometer was used to determine the absorbance at 436 nm of Hb and γ_4_-like at different oxygen partial pressures (PO_2_) (in six parallel replicates). The oxygen saturation (SO_2_) of Hb and γ_4_-like was calculated as follows: SO_2_ $=\frac{\text{A}-\text{A}0}{\text{A}100-\text{A}0}$ , where A_0_ is the absorbance in high-purity nitrogen, A_100_ is the absorbance in air, and A is the absorbance under a certain PO_2_. The relationship between PO_2_ and SO_2_ was fitted with a Hill S-type equation as follows: SO_2_ $=\frac{PO2^{n50}}{P50^{n50}+PO2^{n50}}$ , where P_50_ is the PO_2_ corresponding to half of blood oxygen saturation, and n_50_ is the Hill cooperativity coefficient.

### Determination of Dissolved Oxygen in PS

In order to compare the dissolved oxygen (DO) content in the PS of plateau zokors to that of Sprague-Dawley rats, the freeze-dried PS powder of each sample pool was dissolved in deionized water and the concentration was adjusted to 0.05 g/ml, and every solution was divided into two isovolumetric parts. Using a polarographic Clark-type electrode portable dissolved oxygen detector (JPB-607A Shanghai Yidian Science Instrument Co., Ltd.), the dissolved oxygen contents of one part of the solutions were determined after being aired at 37°C for 5 min. In order to analyze the effects of the γ4-like on the dissolved oxygen content in the PS, the other part of the solutions were ventilated with carbon monoxide (CO) at 37°C for 5 min, and the dissolved oxygen contents were determined by JPB-607A.

### Data Analyses

All values are expressed herein as means ± standard deviation (SD). All statistical analyses consisted of one-way analyses of variance (ANOVAs) followed by Duncan’s post-hoc tests, which were completed using SPSS 22.0 (SPSS Inc., Chicago, IL, USA). *p* < 0.05 was considered statistically significant. All the charts were visualized by origin 9.0, and all drawings were made in CorelDraw X8.

## Results

### The Characteristics of the Heme-ContainingProtein in the PS of Plateau Zokor

**FIGURE 1:**
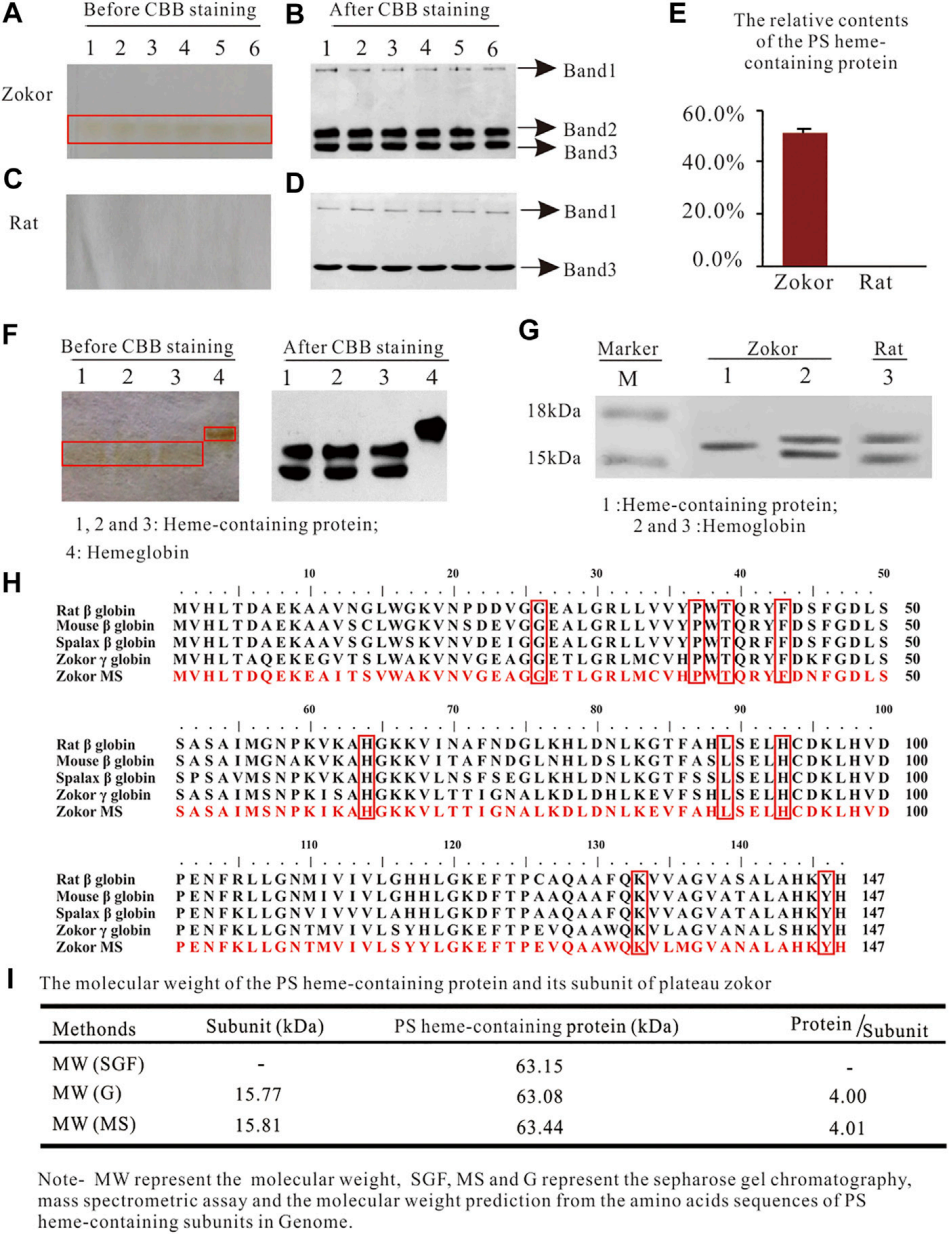
The characteristics of the heme-containing protein in the PS of plateau zokors. **(A-D)** The PAGE gel electrophoresis of proteins in the PS solution of plateau zokors **(A,B)** and Sprague-Dawley rats **(C,D)** before/after Coomassie brilliant blue (CBB) staining (*n* = 6). **(E)** The relative content of the PS heme-containing protein. **(F)** The PAGE gel electrophoresis of proteins in the PS solution and the hemoglobin (Hb) before/after CBB staining. **(G)** The SDS-PAGE results of the PS heme-containing protein and Hb (*n* = 6). **(H)** The homology alignments of the AAs among the PS heme-containing subunit measured by mass spectrometric assay (Zokor MS) and the adult major β globins of other four rodents downloaded from GenBank databases (the conserved AAs were labeled in red box). **(I)** The molecular weight of the heme-containing protein measured by sepharose gel chromatography and that of its subunit predicted by ProtParam tool according to its AAs in the genome and confirmed by MS, respectively.

**FIGURE 2:**
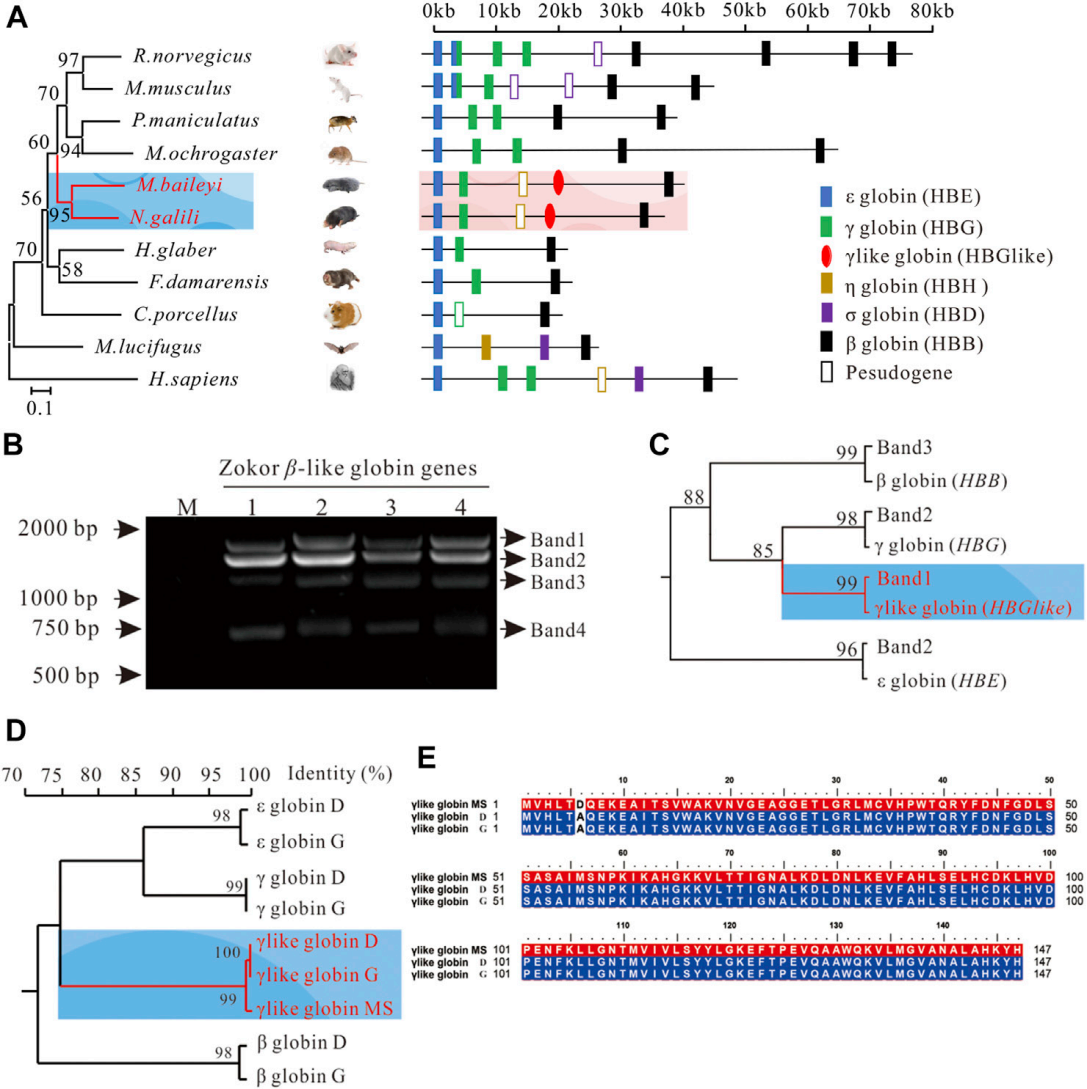
Identification of the gene encoding the γ-like subunit of plateau zokor. **(A)** The genomic structures of the *β*-globin gene clusters in 11 studied placental mammals. Diagonal slashes indicate gaps in genomic coverage. Segments containing such gaps were not drawn to scale. The orientation of the cluster is from 5′ (on the left) to 3′ (on the right). Phylogenetic tree of mtDNA of 11 mammals, inferred by Bayesian inference under the GTR + I + G model. The branch length is shown and the posterior probabilities values (%) are indicated on the nodes. **(B)** The bands of the cloning *β*-like globin genes in the lung tissue of plateau zokors by agarose gel electrophoresis. Band-1, -2, -3, and -4 represent the *β*-like globin genes in the lung tissue of plateau zokors by agarose gel electrophoresis (*n* = 4). **(C)** Maximum likelihood phylograms depicting relationships among *β*-like globin genes determined by DNA sequencing (Band-1, -2, and -3) and annotated from the *β*-globin gene cluster by Genescan and blast2. Bootstraps support for the relevant nodes was evaluated using 1,000 pseudoreplicates. The tree demonstrated that band-1 and -3 is γ-like and *β* globin and band-2 contains γ and ε globin. **(D)** The homology tree of the *β*-like globins’ AAs translated from the CDS by DNA sequencing, annotated from the β-globin gene cluster, and the AAs detected by the MS assay. The tree was constructed by DNAMAN version 9.0. **(E)** The homology alignments among the γ-like globin sequence detected by MS (γ-like globin MS), translated from the CDS by DNA sequencing (γ-like globin D) and annotated from the β-globin gene cluster of plateau zokor (γ-like globin G).

Using PAGE, we observed a red protein band in the PS of plateau zokors before CBB staining (Figure 1A)but not in that of Sprague-Dawley rats (Figure 1C). After CBB staining, we observed three and two protein bands in the PS of plateau zokors and Sprague-Dawley rats, respectively (Figures 1B,D). The band 2 observed in the PS of plateau zokors after CBB staining showed the same electrophoretic mobility as the red protein band before CBB staining (Figures 1A,B). Therefore, the protein band 2 is aheme-containingprotein. Theheme-containingprotein constitutes about 54.51% ± 1.30% of the total PS proteins (Figure 1E) and showed a different electrophoretic mobility from the Hb of plateau zokors (Figure 1F). Using SDS-PAGE, we observed the heme-containing protein consists of a single subunit, whereas the Hb of plateau zokors has two subunits (Figure 1G). The subunit of the heme-containing protein has 147 amino acids determined by mass spectrometric assay (MS). It has nine identical conserved amino acids with the Hb *β*-like subunits of mouse, rat, spalax and plateau zokor itself, namely 26 Gly (B6), 37Pro (C2), 39 Thr (C4), 46Phe (CD1), 64His (E7), 89Leu (F4), 93His (F8), 133Lys (H10), and 146Tyr (HC2) (Figure 1H), and the AAs of the subunit shared 93.90%, 70.10%, and 70.10% identity with that of the γ, ε, and β subunit of plateau zokor, respectively (Figure 2D). The molecular weight of the heme-containing protein is 63.15 kDa by sepharose gel chromatography and that of its subunit is 15.81 kDa, as determined by MS (Figure 1I). The relative molecular weight of the protein is approximately four times greater than that of its subunit. Therefore, the heme-containing protein is a homotetramer hemoglobin which is constituted by four γ-like subunits. According to the nomenclature of Aguileta et al. (2006) (Aguileta et al., 2006), we named the new heme-containing protein as γ_4_-like.

### Identification of the Gene Encoding the γ-Like Subunit of Plateau Zokor

To identify the gene that encodes the γ-like subunit, we first obtained the genomic sequences spanning the β-globin gene cluster of the plateau zokor (Supplementary Figure S1, https://doi.org/10.6084/m9.figshare.13186766.v1). Using Genescan and blast2, we found that the size of the *β*-globin gene cluster of plateau zokor is about 40 kb and the structure is *5′-ε-γ-ψη-γlike-β-3′* (Figure 2A, Supplementary Figure S1, https://doi.org/10.6084/m9.figshare.13186766.v1). Although the size and membership compositions of the gene family among the 11 selected species (plateau zokor, *Myospalax baileyi*, African naked mole rat, *Heterocephalus glaber*, Northern Israeli blind subterranean mole rat, *Nannospalax galili*, Sprague-Dawley rat, *Rattus norvegicus*, mouse, *Mus musculus*, Damaraland mole rat, *Fukomys damarensis*, Guinea pig, *Cavia porcellus*, Deer mouse, *Peromyscus maniculatus*, Prairie voles, *Microtus ochrogaster*, little brown bat, *Myotis lucifugus* and human, *Homo sapiens*) showed obvious variation, it is identical between the plateau zokor and the Middle East mole rat, *Spalax ehrenbergi* (Figure 2A), which is also a fossorial rodent living in sealed hypoxic burrows. Then, we extracted the DNA from the intensively perfused lung tissues of plateau zokor, using degenerate primers (Figure 2B, Supplementary Figures S2, S3, Supplementary Figure S2, https://doi.org/10.6084/m9.figshare.13186766.v1); four bands of the *β*-like globin genes were cloned and sequenced. The maximum-likelihood tree between the *β*-like globin genes from genomic sequences and our PCR cloning sequences demonstrated that band-1, -3, and -4 correspond to *γ-like* (1,354 bp), *β* (1,156 bp), and *ψη* (752 bp), respectively, and band-2 includes *ε* (1,245 bp) and *γ* (1,262 bp) (Figures 2B,C, Supplementary Figure S3, Supplementary Figure S2, https://doi.org/10.6084/m9.figshare.13186766.v1). Last, by homology alignments, we found that the coding sequence (CDS) of the γ-like and the AAs of the γ-like subunit of plateau zokor shared the highest identities with that of itself *γ* and γ subunit, respectively (Figure 2D, Supplementary Figure S4, Supplementary Tables S1, S2, https://doi.org/10.6084/m9.figshare.13186766.v1). The AAs of *γ-like* globin determined by MS shared 99.30% identity with that annotated from the *β*-globin gene cluster of plateau zokor (Figure 2E, Supplementary Figure S5, Supplementary Tables S1, S2, https://doi.org/10.6084/m9.figshare.13186766.v1). Therefore, we confirmed that the subunit of the homotetramer hemoglobin in the PS of plateau zokor is encoded by *γ-like*.

### The Origination, Structure, Tissue-Specific Expression and Response to Hypoxia of *γ-Like*

**FIGURE 3:**
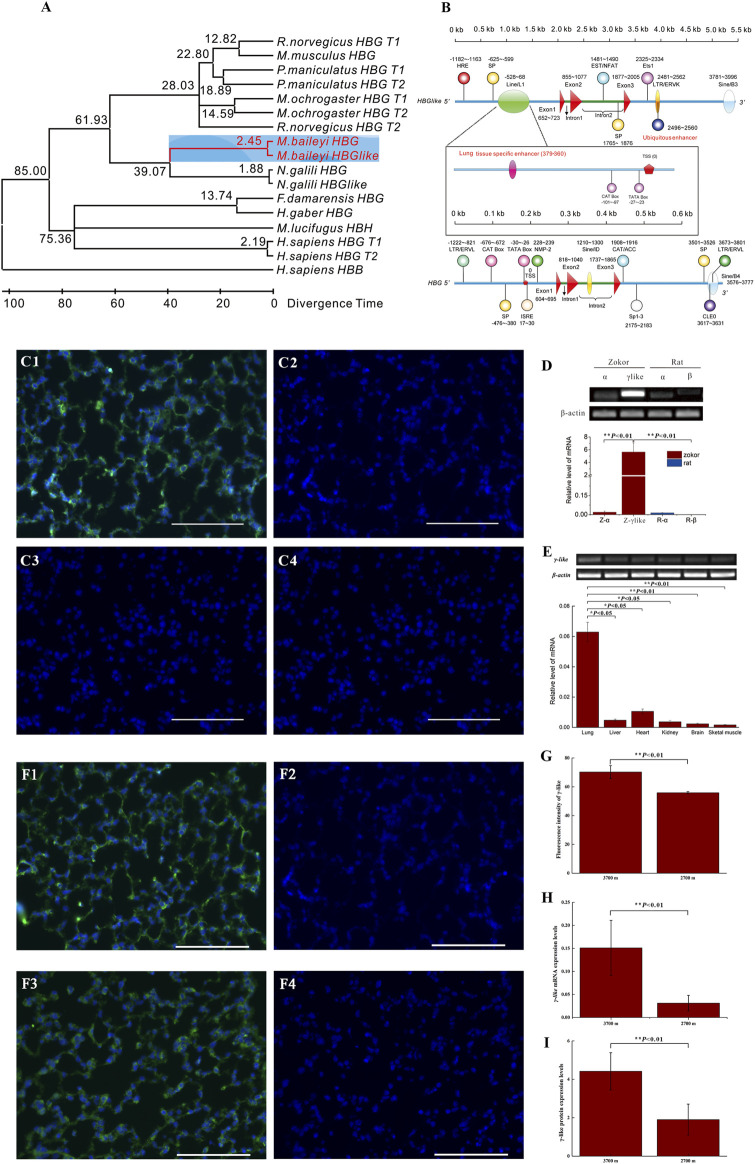
The structure, tissue-specific expression, and response to hypoxia of *γ-like*. **(A)** Maximum-likelihood tree of the *γ-like* and *γ* coding sequences of eight studied species. The divergence time was predicted by MEGA7.0 program and is shown on each node. The range of Euarchontoglires-Laurasiatheria divergence times spanning about 85 Mya was used as a calibration point. **(B)** The structures of *γ-like* and *γ* of plateau zokor. The whole structures contain 2 kb of 5′ flanking sequence, three exons, two introns, and 2 kb of 3′ flanking sequence. The promoters, repeats, transposons, and transcription regulatory elements were indicated. **(C1–C4)** Immunofluorescence staining for γ-like subunit in lung tissues of plateau zokor and Sprague-Dawley rat (C1, C2**-**plateau zokor, C3, C4**-**Sprague-Dawley rat). **(C2,C4)** Negative controls. Objective magnification: ×40, scale bar = 100 μm. The DAPI labeled the nucleus blue, the γ-like subunit was labeled green. **(D)** The mRNA expression levels of α, β, and γ-like by qRT-PCR in lung tissues of plateau zokors and Sprague-Dawley rats, respectively (*n* = 8). Z, plateau zokor. R, Sprague-Dawley rat. **(E)** The expression levels of γ-like in the lung, liver, heart, kidney, brain, and skeletal muscle tissues of plateau zokor by qRT-PCR *(n* = 8). **(F1–F4)** Immunohistochemistry patterns for γ-like subunit in lung tissues of plateau zokors (F1, F2**-**3,700 m, F3, F4**-**2,700 m), (*n* = 8). **(F2,F4)** Negative controls. **(G)** The statistics results of the fluorescence intensity of γ-like subunit in the lung of plateau zokors. **(H,I)** The expression levels of γ-like by qRT-PCR **(H)** and γ-like subunit by Western blot **(I)** at altitude 3,700 and 2,700 m in lung tissues of plateau zokor (*n* = 8). *p < 0.05, **p < 0.01.

The Bayesian tree based on the upstream and downstream flanking sequences and intron 2 sequences indicated that *γ-like* is a paralogous gene of *γ* (Supplementary Figures S5, S6, Supplementary Figure S3, https://doi.org/10.6084/m9.figshare.13186766.v1). The Bayesian tree based on the coding sequences (three exons) indicated that the *γ-like* was differentiated from *γ* gene through duplication without conversions between *γ-like* and *γ* (Supplementary Figures S4, S5, https://doi.org/10.6084/m9.figshare.13186766.v1). The *γ-like* is 92 bp longer than *γ,* and the two subunits have nine different amino acids (Supplementary Figures S3, S4, https://doi.org/10.6084/m9.figshare.13186766.v1)*.* Using the MEGA 7.0 program, we analyzed the divergence time between *γ-like* and *γ*. Our results showed that the divergence times of the plateau zokor’s *γ-like* was estimated by the molecular clock to be about 2.45 Mya, i.e., between the late Pliocene and the early Quaternary (3.6–2.5 Mya) (Figure 3A). Using the Nsite and GPminer online software, the promoters and transcription regulatory elements (TREs) of plateau zokor’s *γ-like* and *γ* were predicted. The results showed that the *γ-like* and *γ* of plateau zokor have 12 and 13 TREs, respectively, distributed over the 2 kb upstream, intron 2, and 2 kb downstream of the gene sequences (Supplementary Figure S6, https://doi.org/10.6084/m9.figshare.13186766.v1). Using the GeneCards databases to annotate the TREs, we found that the TREs are clearly different between the *γ-like* and *γ*. Notably, unlike *γ,* the *γ-like* has a hypoxia response element (HRE) in its upstream region and two enhancers in its upstream and downstream regions, respectively (Figure 3B, Supplementary Figure S6, Supplementary Tables S3, S4, https://doi.org/10.6084/m9.figshare.13186766.v1). The HRE of the *γ-like* contains two critical elements, namely hypoxia-inducible factor 1 (HIF-1) binding sites (HBS, core sequence ACGTGC) and a HIF-1 ancillary sequence (HAS, core sequence CAGGT) (Figure 3B, Supplementary Figure S6, Supplementary Table S3, https://doi.org/10.6084/m9.figshare.13186766.v1). By mapping the sequence of the two enhancers to the human Tissue-specific Enhancer Database (TiED), we revealed that the enhancer upstream of the *γ-like* of the plateau zokor is a lung tissue-specific enhancer and the enhancer downstream of the *γ-like* is a ubiquitous enhancer. (TiED id, GO:0051059/enh_9877) (Supplementary Tables S5–S9, https://doi.org/10.6084/m9.figshare.13186766.v1).

Immunofluorescence demonstrated that the γ-like subunit was expressed obviously in lung tissue of plateau zokors, but not in that of Sprague-Dawley rats (Figures 3C1–C4). The mRNA of γ-like was significantly expressed, specifically in lung tissues of plateau zokors, hardly in that of Sprague-Dawley rats (*p* < 0.05) (Figure 3D) and in liver, heart, kidney, brain, and skeletal muscle tissues of plateau zokor (*p* < 0.05) (Figure 3E). In lung tissues of plateau zokors, the expression levels of γ-like mRNA and protein were significantly higher in the high-altitude group (3,700 m) than those in the low-altitude group (2,700 m) (*p* < 0.01) (Figures 3F–I).

### The Expression of γ-Like Subunit in ATI, ATII, and LBs of Plateau Zokor

**FIGURE 4:**
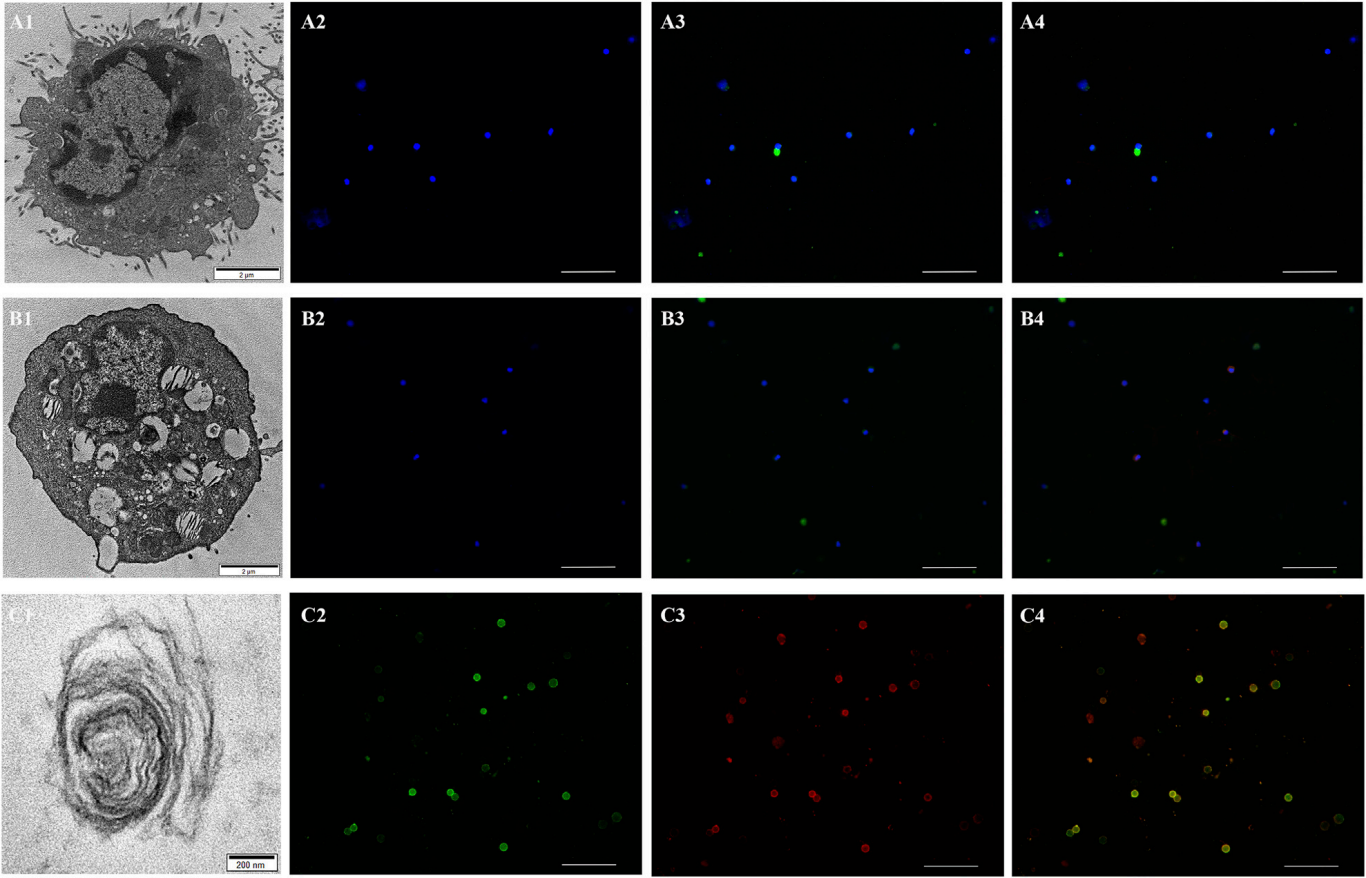
The expression of γ-like subunit in ATI, ATII and LBs of plateau zokor. **(A1,B1,C1)** Transmission electron microscopy (TEM) patterns of the ATI **(A1)**, ATII **(B1)** , and LB **(C1)** of plateau zokors. **(A2,B2)** The controls of the ATI **(A2)** and ATII **(B2)** of plateau zokors. **(A3,B3)** Immunofluorescence staining of the AQP-5 in ATI (A3, green) and SP-C in ATII (B3, green) of plateau zokors. **(A4,B4)** Immunofluorescence staining of the γ-like subunit in ATI (A4, green + red) and ATII (B4, green + red) of plateau zokors. The DAPI labeled the nucleus blue; objective magnification: ×20, scale bar = 100 μm. **(C2,C3,C4)** Immunofluorescence staining of the SP-C (C2, green), γ-like subunit (C3, red), and SP-C + γ-like subunit (C4, green + red) in LBs of plateau zokors. Objective magnification: ×40, scale bar = 100 μm.

By using the Flow Cytometric Cell Sorting and AQP-5 as the cell-specific marker of ATI and SP-C as that of ATII and LBs, the ATI, ATII, and LBs of plateau zokors were separated and collected. We clearly observed the structurally intact ATI (Figure 4A1), ATII (Figure 4B1) and LBs (Figure 4C1) by transmission electron microscopy. Using AQP-5 and SP-C antibodies, we tested the purities of ATI and ATII cells by immunofluorescence cross validation; the results showed that the purities of ATI and ATII cells were 100.00% and 95.00%, respectively. Using the *γ-like* antibody, we verified that the *γ-like* was expressed in ATII (Figure 4B4) and LBs (Figure 4C4), not in ATI of plateau zokors (Figure 4A4).

### The Oxygen Dissociation Curve of γ_4_-Like and Its Effect on the Dissolved Oxygen Content in the PS of Plateau Zokors

**FIGURE 5:**
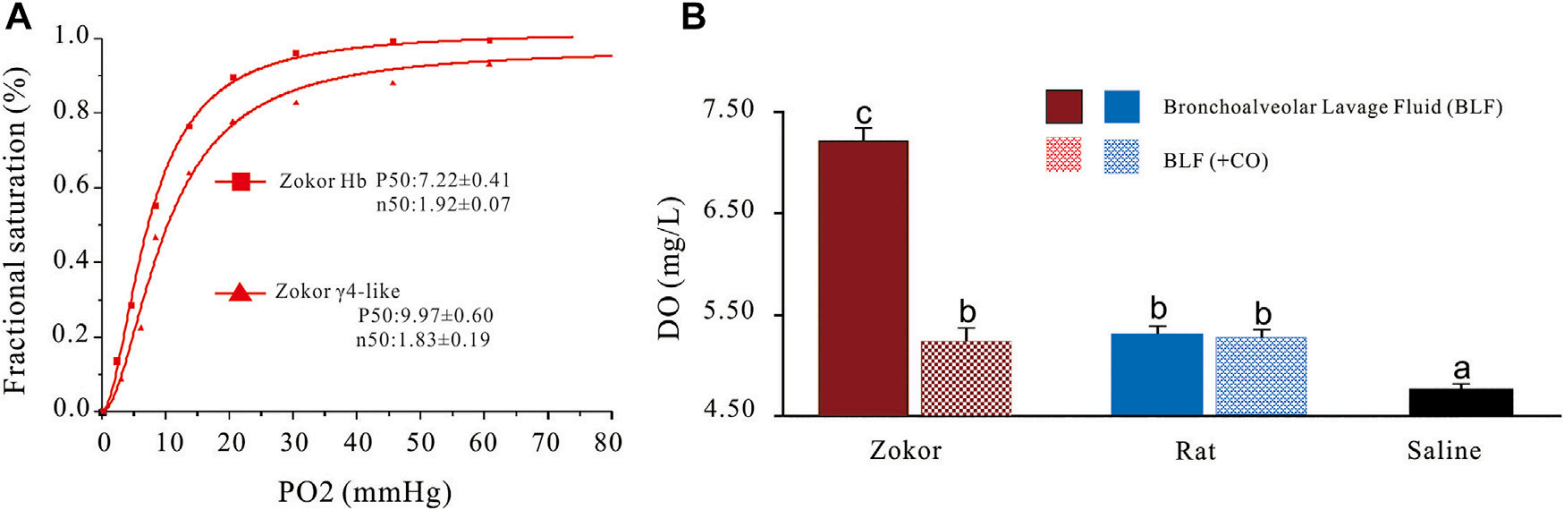
The oxygen dissociation curve of γ_4_-like and its effect on the dissolved oxygen content in the PS of plateau zokors. **(A)** The oxygen dissociation curves of γ_4_-like and Hb of plateau zokor. **(B)** The dissolved oxygen contents in the PS solutions of plateau zokors and SD rats after being aired and ventilated with carbon monoxide (CO) for 5 min (*n* = 6). The saline was used as control group.

The oxygen dissociation curve demonstrated that the γ_4_-like in the PS of plateau zokor had higher P_50_ compared to that of its hemoglobin; the affinity of γ_4_-like to oxygen is lower than that of its hemoglobin (Figure 5A). When the PS solutions were aired for 5 min, the dissolved oxygen (DO) in plateau zokors and Sprague-Dawley rats was 51.47 and 13.03% higher than that in saline, respectively (*p* < 0.01), and that in the PS solutions of plateau zokors was 34.01% higher than that in Sprague-Dawley rats (*p* < 0.05) (Figure 5B). When the PS solutions of plateau zokors and Sprague-Dawley rats were ventilated with carbon monoxide (CO) for 5 min, the DO in plateau zokors was significantly decreased by 28.43% (*p* < 0.01), and it had no significant decrease in that of Sprague-Dawley rats (Figure 5B) (*p* > 0.05).

## Discussion

The plateau zokor (*Myospalax baileyi*) is a fossorial rodent that spends its entire life underground in sealed hypoxic burrows at altitudes of 2,800 to 4,200 m (Fan and Shi, 1982). The average oxygen concentration in the burrows of plateau zokors is found to be 20% lower than that of the local atmospheres (Zeng et al., 1984). Over evolutionary time, the plateau zokor has developed a series of physiological and molecular mechanisms for effective oxygen absorption, transfer, and utilization (Zeng et al., 1984; Widmer et al., 1997; Wei et al., 2006). Recent studies showed that the genes involved in respiratory gaseous exchange are significantly positively selected in the plateau zokor (Shao et al., 2015); this adaptation might be a benefit to absorb oxygen from the severe hypoxic environments.

The lung is a gas exchange organ (Lefrançais et al., 2017). Alveolar epithelium of the lung is primarily composed of type I cells (ATI) and type II cells (ATII) (Crandall and Matthay, 2001). PS is synthesized by ATII pneumocytes and secreted into the alveolar cavities through the osmiophilic multilamellar body (LBs). PS is composed mainly of phospholipids and proteins which have functions of facilitating respiratory gas exchange by increasing oxygen-dissolving capability and oxygen diffusion in the pulmonary surfactant (Olmeda et al., 2010). Up to date, it is widely believed that the protein moiety of surfactant comprises four specific surfactant-associated proteins (Haagsman and Diemel, 2001). They can be classified in two groups, the hydrophilic surfactant proteins SP-A and SP-D, and the hydrophobic surfactant proteins SP-B and SP-C (Serrano and Pérez-Gil, 2006). In the present paper, our study results showed that a homotetramer hemoglobin composed of γ-like subunit existing in the PS of plateau zokors, but not in that of Sprague-Dawley rats. The homotetramer hemoglobin constituted about 54.51% ± 1.30% of the total PS proteins. At present, the homologous tetramer hemoglobin (γ_4_ or β_4_) has not been found in mammals, except for γ_4_^F^ (in the fetal stage) and β_4_^S^ (in the adult stage) in erythroid cells of human α-thalassemias, because of the decreased levels of the *α*-globin gene mRNA (Jones et al., 1959). To our knowledge, it is a unique finding that provides a new insight into the compositions of proteins in the PS of mammals and could potentially have important implications for the physiology of the lung.

Hemoglobins are heterotetramer proteins consisting of two α-globin-like and two *β*-globin-like chains (Hardison, 1996). The α- and *β*-globin gene clusters of mammals contain a set of developmentally regulated genes that are arranged according to their temporal order of expression (Hardison et al., 2001). The structure of the *β*-globin gene cluster in the last common ancestor of mammals is 5′-*ε*-*γ*-*η*-*δ*-*β*-3′ (Hardies et al., 1984). The size of the *β*-globin gene clusters and membership composition of the gene family among the extant eutherian species show considerable variation (Wildman et al., 2007). The differences detected in these genes among species are primarily attributable to lineage-specific gene gains *via* duplication and lineage-specific losses *via* deletion or inactivation, which resulted in differences in the structure of the genomic region, expression patterns, and physiological functions between (or among) paralogous genes (Opazo et al., 2008). In the present paper, we obtained the genomic sequences spanning the *β*-globin gene cluster of the plateau zokor. Using Genescan and blast2, we found that the size of the *β*-globin gene cluster of the plateau zokor is about 40 kb and the structure is *5′-ε-γ-ψη-γlike-β-3′*. Although the size and membership compositions of the gene family among the 11 species show obvious variation, they are identical between the plateau zokor and the Middle East mole rat, *Spalax ehrenbergi*, which is also a fossorial rodent living in sealed hypoxic burrows (Arieli et al., 1977). We confirmed that the subunit of the homotetramer hemoglobin in the PS of plateau zokor is encoded by the *γ-like* of plateau zokor’s *β*-globin gene cluster; the *γ-like* is a 1:1 paralogous gene of *γ*. However, the *γ-like* is 92 bp longer than *γ,* and the two subunits have nine different amino acids*.* Therefore, the lineage-specific *γ-like* of plateau zokors is a paralogous gene of *γ*.

The ancestral population of plateau zokors (*Prosiphneus* Teilhard de Chardin, 1926) was composed of surface-dwelling rodents living in warm and humid environments distributed in North China, including the Loess Plateau in the middle Miocene (11.7 Mya) (Zheng et al., 2004). Using the MEGA 7.0 program, we analyzed the divergence time between *γ-like* gene and *γ* of plateau zokor; our results showed that the divergence times of the *γ-like* gene was estimated by the molecular clock to be about 2.45 Mya, i.e., it was in the time of late Pliocene and the early Quaternary (3.6–2.5 Mya). During this period, with the rapid uplift of the Qinghai-Tibet Plateau (which occurred 3.4 Mya ago) and the beginning of the Quaternary glacial cycles, the climate in the region began to undergo a succession from a tropical and subtropical climate to a cold and dry climate (Liu, 1988; Zhang et al., 2000). Incapable of tolerating the cold and dry environments, the *Prosiphneus* had to move to the underground and conquer extremely hypoxic and hypercapnia burrows. The population gradually spread from the east Qinling Mountains to the west Qinghai-Tibet Plateau and differentiated into the plateau zokors. After the Quaternary Ice Age, with further uplift of the Qinghai-Tibet Plateau, the oxygen concentrations in the burrows of plateau zokors became lower. Studies revealed that the Middle East mole rat, *Spalax ehrenbergi*, which is found in Israeli Golan Heights with an average altitude about 500 m, survives at O_2_ tensions below those at the summit of Mount Everest and CO_2_ tensions 200-fold higher than in the air above the ground (Shams et al., 2005). Plateau zokors inhabit burrows at elevations of 2,800 to 4,200 m (Fan and Shi, 1982); the O_2_ tensions in the burrows might be lower than those of spalax.

Hypoxia had profound effects on the evolution and function of hemoglobin genes, as the hemoglobin genes of animals that are native to hypoxia environments are modified by specific mutations or differentiated by tandem duplication, and generating functional divergence iso-Hbs that enhance the hypoxia-native species adaptation to hypoxia environments (Lalthantluanga et al., 1985; Hardison and Miller, 1993; Natarajan et al., 2013; Galen et al., 2015). Survivorship studies of free-ranging deer mice demonstrated that the aerobic performance is subject to strong directional selection at high-altitude (Storz et al., 2012). A multi-locus analysis of nucleotide polymorphism and linkage disequilibrium revealed that high-altitude adaptation of the deer mouse hemoglobin involves parallel functional differentiation at multiple unlinked gene duplicates: two α-globin paralogs and two *β*-globin paralogs, which resulted in the differences in O_2_-binding affinity of hemoglobin isoforms between the high-altitude deer mouse and low-altitude mouse (Natarajan et al., 2013). The red blood cells of the Rüppell’s griffon (*Gyps rueppelli*), a high-soaring African vulture, contain a mixture of four distinct α-chain iso-Hbs (HbA, HbA′, HbD, and HbD′) with graded O_2_-binding affinities as a result of the tandem duplication and functional divergence of the α^A^- and α^D^-globin genes; the high-affinity α^D^-chain isoHbs appear to have helped safeguard the arterial O_2_ saturation (Storz and Moriyama, 2008). Under conditions of high-altitude hypoxia, adult alpacas (*Vicugna pacos*) and yaks (*Bos grunniens*) are known to up-regulate a fetal *β*-like globin gene, which results in the synthesis of a relatively high-affinity fetal Hb (Reynafarje et al., 1975; Sarkar et al., 1999; Qiu et al., 2012). In addition to the coexpression of fetal and adult Hbs under hypoxic conditions, yaks also possess multiple adult isoHbs due to functional differentiation among the tandemly duplicated α- and *β*-globin genes (Lalthantluanga et al., 1985; Weber et al., 1988). The cascaded O_2_ affinities of the fetal and adult isoHbs appear to play an important role in the hypoxia tolerance of yak during both pre- and postnatal life (Weber et al., 1988).

Paralogous genes are produced by spot mutations, gene conversion, gene recombination, and multiple insertions of retroposon-type repeats after the last common ancestor gene duplication (Hardison and Miller, 1993; Galen et al., 2015). Using the Nsite and GPminer online software, the promoters and transcription regulatory elements (TREs) of plateau zokors’ *γ-like* gene and *γ* were predicted. Our results show that the *γ-like* gene and *γ* of plateau zokor has 12 and 13 TREs, respectively, distributed over the 2 kb upstream, intron 2, and 2 kb downstream of the gene sequences. Using the GeneCards databases to annotate the TREs, we found that the TREs were clearly different between the *γ-like* gene and *γ*. Notably, unlike *γ,* the *γ-like* gene has a hypoxia response element (HRE) in its upstream region, a lung tissue specific enhancer in its upstream region, and a ubiquitous enhancer in its downstream region. Previous studies found that the HRE and enhancers can significantly enhance its gene expression (Semenza et al., 1991; Levine, 2010). Our qRT-PCR and Western blot analysis results indicated that *α*, *β*, and *γ* genes were hardly expressed, but the *γ-like* gene was highly expressed in the lung tissues of plateau zokors and barely expressed in the other tissues, and its expression was up-regulated by hypoxia.

It has long been thought that hemoglobin itself is expressed only in cells of the erythroid lineage in vertebrates and that the expression of the α- and *β*-globin genes is balanced and coordinated to produce functional hemoglobin heterotetramers consisting of two α-globin-like and two *β*-globin-like chains (Hardison, 1996). The α- and *β*-globin gene clusters of mammals contain a set of developmentally regulated genes that are arranged according to their temporal order of expression (Hardison et al., 2001). However, studies reported that the α- and *β*-globin genes of Hb are expressed extensively in non-erythroid cells, such as lens cells (Wride et al., 2003); murine macrophages (Liu et al., 1999); ATII and Club cells of humans, mice, and rats (Newton et al., 2006; Dassen et al., 2008); neurons of the rodent brain; mesangial cells of the normal rat kidney; and human and murine arterial endothelial cells (enriched at the myoendothelial junction) (Williams, 2003; Bishop, 2004; Nishi et al., 2008; Schelshorn et al., 2009; Straub et al., 2012). However, it has been identified that the globin protein production is not as tightly coordinated in pulmonary epithelial cells as in erythroid cells. Our results show that, of the α- and *β*-globin genes, only the *γ-like* was expressed in lung tissues of plateau zokors, and that is not coordinated with the α-globin genes.

Based on above results, we suggested that, for long-time hypoxia stress, the *γ-like* of plateau zokor was differentiated from *γ via* duplication and spot mutations, and its lung tissue-specific and hypoxia up-regulated expression patterns might well be related to the lung tissue specific enhancer and the HRE. The *γ-like* expression in lung tissues of plateau zokors were not balanced and coordinated with α-globin genes.

Alveolar epithelium of the lung is primarily composed of ATI and ATII cells. The ATI are large squamous cells while ATII are cuboidal in shape (Crandall and Matthay, 2001). ATII are stem cells that undergo significant phenotypic changes to terminally differentiate into ATI (Williams, 2003; Bishop, 2004). Previous studies demonstrated that both the α- and *β*-globin genes of hemoglobin were expressed in ATII cells, but not in ATI cells of human, mouse, and rat. Because of the difference in the functions of these 2 cell types, there are a large number of genes and proteins differentially expressed in these cells, and many of them have been used as markers for the specific cell type (Dobbs et al., 1985; Padró et al., 1990; Kalina et al., 1992). Using AQP-5 as the cell-specific marker of ATI and SP-C as that of ATII and LBs, we separated the ATI, ATII, and LBs of plateau zokors by Flow Cytometric Cell Sorting. The purities of ATI and ATII cells were 93% and 92%, respectively. By transmission electron microscopy, we clearly observed the ATII of plateau zokors with structurally intact LBs and the ATI without LBs. We also verified that the *γ-like* was expressed in ATII and LBs of plateau pikas but not in their ATI. As is well-known, the PS is synthesized in ATII cells and secreted into alveolar spaces through LBs. Therefore, the high concentrations of γ_4_-like in the PS of plateau zokors come from LBs which are secreted into the alveolar cavities by the ATII.

PS complexes are assembled in type II pneumocytes and stored in the form of tightly packed membranes in the LBs which are secreted outside the cells and form multiple interconnected membrane structures including secreted lamellar bodies, tubular myelin, and multilayered films in alveoli airspace (Weaver et al., 2002; Pérez-Gil, 2008). The phospholipid reduces the surface tension at the respiratory air-liquid interface (Serrano and Pérez-Gil, 2006). Both SP-A and SP-D play an important role in the innate immune system (Korfhagen et al., 1996; Crouch and Wright, 2001). In addition to its innate immune function, SP-A is necessary for the formation of tubular myelin which enhances surfactant adsorption (882) and has a synergistic effect in the enhancement of lipid interfacial adsorption promoted by SP-B and SP-C (Schürch et al., 1992; Bi et al., 2001; Possmayer et al., 2001). However, SP-B and SP-C play critical roles in formation and stabilization of pulmonary surfactant films (Weaver and Conkright, 2001). Interestingly, scientists testified that capillary water layers containing enough density of pulmonary surfactant membranes transport oxygen much faster than a pure water phase or a water layer saturated with purely lipidic membranes (Olmeda et al., 2010). Membranes reconstituted from whole pulmonary surfactant organic extract, containing all the lipids plus the hydrophobic surfactant proteins, also permit very rapid oxygen diffusion, substantially faster than achieved by membranes prepared from the surfactant lipid fraction depleted of proteins (Olmeda et al., 2010). Their results also show that the oxygen-diffusion properties of pulmonary surfactant complexes stand beyond the particularly high solubility of dioxygen into phospholipid membranes (Olmeda et al., 2010). Our results showed that the γ_4_-like, the main protein in the PS of plateau zokor, showed higher O_2_ affinity. The dissolved oxygen in that of plateau zokors was 34.01% higher than that in Sprague-Dawley rats. When the PS solutions of plateau zokors were ventilated with carbon monoxide for 5 min, the dissolved oxygen contents in the solutions were significantly decreased, and that in Sprague-Dawley rats had no significant decrease. These results confirmed that the γ_4_-like increased significantly oxygen-dissolving capability of PS of plateau zokor by its oxygen-storing function, which might be likely to increase the oxygen partial pressure in the PS of plateau zokor during inhalation, and that might speed up the oxygen diffusion from alveoli to blood. In addition, because of the γ_4_-like has oxygen bonding properties, a portion of oxygen can be stored in the PS of plateau zokor during inhalation and the oxygen might be dissociated from the γ_4_-like by the Bohr effect and diffused into blood during expiration, so that the γ_4_-like in the PS of plateau zokor might be beneficial for the plateau zokors to obtain oxygen from the severe hypoxia environments by facilitating oxygen diffusion from alveoli to blood.

In conclusion, for long-time adaptation to the hypoxia environments, a new gene, *γ*-*like*, which was confirmed as a gene of the *β*-globin gene cluster, was diverged from *γ* about 2.45 Mya in plateau zokors. Unlike *γ,* the *γ-like* has a hypoxia response element and a lung tissue-specific enhancer in its upstream region, and it is expressed specifically in lung tissue of plateau zokors and up-regulated by hypoxia. The protein encoded by *γ-like* is a homotetramer hemoglobin which was synthesized in ATII and secreted into the alveolar cavities through LBs as a main protein in the PS of plateau zokors. The protein had oxygen-storing function and increased significantly the oxygen-dissolving capability in the PS of plateau zokor, which might be beneficial for the plateau zokor to obtain oxygen from the severe hypoxia environments by facilitating oxygen diffusion from alveoli to blood. Our unique findings provide new insights into the adaptive mechanisms of plateau zokor to hypoxia, the lineage-specific structure of plateau zokor’s *β*-globin gene cluster, and the compositions of proteins in the PS of mammals, and could potentially have important implications for the physiology of the lung.

## Data Availability

The data presented in the study are deposited in the NCBI (www.nlm.nih.gov), accession number: SRR17794669.

## References

[B1] Aguileta G. Bielawski J. P. Yang Z. (2006). Proposed Standard Nomenclature for the .ALPHA.- and .BETA.-globin Gene Families. Genes Genet. Syst. 81 (5), 367–371. 10.1266/ggs.81.367 17159299

[B2] Arieli R. Ar A. Shkolnik A. (1977). Metabolic Responses of a Fossorial Rodent (*Spalax Ehrenbergi*) to Simulated Burrow Conditions. Physiol. Zoolog. 50 (1), 61–75. 10.1086/physzool.50.1.30155716

[B3] Bi X. Taneva S. Keough K. M. W. Mendelsohn R. Flach C. R. (2001). Thermal Stability and DPPC/Ca2+ Interactions of Pulmonary Surfactant SP-A from Bulk-phase and Monolayer IR Spectroscopy. Biochemistry 40 (45), 13659–13669. 10.1021/bi011188h 11695915

[B4] Bishop A. E. (2004). Pulmonary Epithelial Stem Cells. Cell Prolif 37 (1), 89–96. 10.1111/j.1365-2184.2004.00302.x 14871239 PMC6495778

[B5] Bouton G. D. (2011). CorelDRAW X5 the Official Guide. Tata McGraw-Hill Education.

[B6] Burge C. Karlin S. (1997). Prediction of Complete Gene Structures in Human Genomic DNA. J. Mol. Biol. 268 (1), 78–94. 10.1006/jmbi.1997.0951 9149143

[B7] Chen J. Chen Z. Narasaraju T. Jin N. Liu L. (2004). Isolation of Highly Pure Alveolar Epithelial Type I and Type II Cells from Rat Lungs. Lab. Invest. 84 (6), 727–735. 10.1038/labinvest.3700095 15077123

[B8] Collins F. S. Weissman S. M. (1984). The Molecular Genetics of Human Hemoglobin. Prog. Nucleic Acid Res. Mol. Biol. 31, 315–465. 10.1016/s0079-6603(08)60382-7 6397774

[B9] Crandall E. D. Matthay M. A. (2001). Alveolar Epithelial Transport. Am. J. Respir. Crit. Care Med. 163 (4), 1021–1029. 10.1164/ajrccm.163.4.2006116 11282783

[B10] Crouch E. Wright J. R. (2001). Surfactant Proteins A and D and Pulmonary Host Defense. Annu. Rev. Physiol. 63 (1), 521–554. 10.1146/annurev.physiol.63.1.521 11181966

[B11] Danielsson J. de Haan L. Peng L. de Vries C. G. (2001). Using a Bootstrap Method to Choose the Sample Fraction in Tail index Estimation. J. Multivariate Anal. 76 (2), 226–248. 10.1006/jmva.2000.1903

[B12] Darriba D. Taboada G. L. Doallo R. Posada D. (2012). jModelTest 2: More Models, New Heuristics and Parallel Computing. Nat. Methods 9 (8), 772. 10.1038/nmeth.2109 PMC4594756 22847109

[B13] Dassen H. Kamps R. Punyadeera C. Dijcks F. De Goeij A. Ederveen A. (2008). Haemoglobin Expression in Human Endometrium. Hum. Reprod. 23 (3), 635–641. 10.1093/humrep/dem430 18216035

[B14] Dobbs L. G. Williams M. C. Brandt A. E. (1985). Changes in Biochemical Characteristics and Pattern of Lectin Binding of Alveolar Type II Cells with Time in Culture. Biochim. Biophys. Acta (Bba) - Mol. Cel Res. 846 (1), 155–166. 10.1016/0167-4889(85)90121-1 3839418

[B15] Efstratiadis A. Posakony J. W. Maniatis T. Lawn R. M. O'Connell C. Spritz R. A. (1980). The Structure and Evolution of the Human β-globin Gene Family. Cell 21 (3), 653–668. 10.1016/0092-8674(80)90429-8 6985477

[B16] Fan N. C. Shi Y. Z. (1982). A Revision of the Zokors of Subgenus. Acta Theriologica Sinica 2 (2), 183–199. 10.16829/j.slxb.1982.02.009

[B17] Fehrenbach H. (2001). Alveolar Epithelial Type II Cell: Defender of the Alveolus Revisited. Respir. Res. 2 (1), 33. 10.1186/rr36 11686863 PMC59567

[B18] Galen S. C. Natarajan C. Moriyama H. Weber R. E. Fago A. Benham P. M. (2015). Contribution of a Mutational Hot Spot to Hemoglobin Adaptation in High-Altitude Andean House wrens. Proc. Natl. Acad. Sci. USA 112 (45), 13958–13963. 10.1073/pnas.1507300112 26460028 PMC4653164

[B19] Gamerman D. Lopes H. F. (2006). Markov Chain Monte Carlo: Stochastic Simulation for Bayesian Inference. Boca Raton: CRC Press.

[B20] Ge R.-L. Cai Q. Shen Y.-Y. San A. Ma L. Zhang Y. (2013). Draft Genome Sequence of the Tibetan antelope. Nat. Commun. 4 (1), 1858. 10.1038/ncomms2860 23673643 PMC3674232

[B21] Goerke J. (1998). Pulmonary Surfactant: Functions and Molecular Composition. Biochim. Biophys. Acta 1408 (2-3), 79–89. 10.1016/s0925-4439(98)00060-x 9813251

[B22] Haagsman H. P. Diemel R. V. (2001). Surfactant-associated Proteins: Functions and Structural Variation. Comp. Biochem. Physiol. A: Mol. Integr. Physiol. 129 (1), 91–108. 10.1016/s1095-6433(01)00308-7 11369536

[B23] Hardies S. C. Edgell M. H. Hutchison C. A. (1984). Evolution of the Mammalian Beta-Globin Gene Cluster. J. Biol. Chem. 259 (6), 3748–3756. 10.1016/s0021-9258(17)43158-9 6706976

[B24] Hardison R. Miller W. (1993). Use of Long Sequence Alignments to Study the Evolution and Regulation of Mammalian Globin Gene Clusters. Mol. Biol. Evol. 10 (1), 73–102. 10.1093/oxfordjournals.molbev.a039991 8383794

[B25] Hardison R. C. (1996). A Brief History of Hemoglobins: Plant, Animal, Protist, and Bacteria. Proc. Natl. Acad. Sci. 93 (12), 5675–5679. 10.1073/pnas.93.12.5675 8650150 PMC39118

[B26] Hardison R. (2001). “Organization, Evolution, and Regulation of the Globin Genes,”. Disorders of Hemoglobin: Genetics, Pathophysiology, and Clinical Management. Editors Steinberg M. Forget B. Higgs D. Nagel R. (Cambridge, UK): Cambridge Univ Press), 95–115.

[B27] Hoffmann F. G. Opazo J. C. Storz J. F. (2008). New Genes Originated via Multiple Recombinational Pathways in the -Globin Gene Family of Rodents. Mol. Biol. Evol. 25 (12), 2589–2600. 10.1093/molbev/msn200 18780876 PMC2721551

[B28] Huelsenbeck J. P. Ronquist F. (2001). MRBAYES: Bayesian Inference of Phylogenetic Trees. Bioinformatics 17 (8), 754–755. 10.1093/bioinformatics/17.8.754 11524383

[B29] Imai K. (1982). Allosteric Effects in Haemoglobin. Cambridge, New York: Cambridge University Press.

[B30] Jones R. T. Schroeder W. A. Balog J. E. Vinograd J. R. (1959). Gross Structure of Hemoglobin H. J. Am. Chem. Soc. 81 (12), 3161. 10.1021/ja01521a080

[B31] Kalina M. Mason R. J. Shannon J. M. (1992). Surfactant Protein C Is Expressed in Alveolar Type II Cells but Not in Clara Cells of Rat Lung. Am. J. Respir. Cel Mol Biol 6 (6), 594–600. 10.1165/ajrcmb/6.6.594 1591008

[B32] Kalyaanamoorthy S. Minh B. Q. Wong T. K. F. von Haeseler A. JermiinModelFinder L. S. (2017). ModelFinder: Fast Model Selection for Accurate Phylogenetic Estimates. Nat. Methods 14 (6), 587–589. 10.1038/nmeth.4285 28481363 PMC5453245

[B33] Katoh K. Standley D. M. (2013). MAFFT Multiple Sequence Alignment Software Version 7: Improvements in Performance and Usability. Mol. Biol. Evol. 30, 772–780. 10.1093/molbev/mst010 23329690 PMC3603318

[B34] Kingma P. S. Whitsett J. A. (2006). In Defense of the Lung: Surfactant Protein A and Surfactant Protein D. Curr. Opin. Pharmacol. 6 (3), 277–283. 10.1016/j.coph.2006.02.003 16580255

[B35] Korfhagen T. R. Bruno M. D. Ross G. F. Huelsman K. M. Ikegami M. Jobe A. H. (1996). Altered Surfactant Function and Structure in SP-A Gene Targeted Mice. Proc. Natl. Acad. Sci. 93 (18), 9594–9599. 10.1073/pnas.93.18.9594 8790375 PMC38473

[B36] Kumar S. Stecher G. Tamura K. (2016). MEGA7: Molecular Evolutionary Genetics Analysis Version 7.0 for Bigger Datasets. Mol. Biol. Evol. 33 (7), 1870–1874. 10.1093/molbev/msw054 27004904 PMC8210823

[B37] Lalthantluanga R. Wiesner H. Braunitzer G. (1985). Studies on Yak Hemoglobin(Bos grunniens,Bovidae): Structural Basis for High Intrinsic Oxygen Affinity. Biol. Chem. Hoppe-Seyler 366 (1), 63–68. 10.1515/bchm3.1985.366.1.63 4005038

[B38] Lefrançais E. Ortiz-Muñoz G. Caudrillier A. Mallavia B. Liu F. Sayah D. M. (2017). The Lung Is a Site of Platelet Biogenesis and a Reservoir for Haematopoietic Progenitors. Nature 544 (7648), 105–109. 10.1038/nature21706 28329764 PMC5663284

[B39] Levine M. (2010). Transcriptional Enhancers in Animal Development and Evolution. Curr. Biol. 20 (17), R754–R763. 10.1016/j.cub.2010.06.070 20833320 PMC4280268

[B40] Li M. Tian S. Jin L. Zhou G. Li Y. Zhang Y. (2013). Genomic Analyses Identify Distinct Patterns of Selection in Domesticated Pigs and Tibetan Wild Boars. Nat. Genet. 45 (12), 1431–1438. 10.1038/ng.2811 24162736

[B41] Li Y. X. Xu B. An Z. F. Wang Z. J. Li J. M. Gao C. H. (2021). Comparison of the Composition and Content of Pulmonary Surfactant Among Plateau Zokors, Plateau Pikas and Rats. Sheng Li Xue Bao 73 (1), 51–61. 10.13294/j.aps.2020.0060 33665660

[B42] Liu K.-b. (1988). Quaternary History of the Temperate Forests of China. Quat. Sci. Rev. 7 (1), 1–20. 10.1016/0277-3791(88)90089-3

[B43] Liu L. Zeng M. Stamler J. S. (1999). Hemoglobin Induction in Mouse Macrophages. Proc. Natl. Acad. Sci. 96 (12), 6643–6647. 10.1073/pnas.96.12.6643 10359765 PMC21968

[B44] Liu R. H. (1995). Classification and Geographic Zoning of Chinese Zokor. Territory Nat. Resour. Study 3, 54–56.

[B45] Liu W. Xie Y. Ma J. Luo X. Nie P. Zuo Z. (2015). IBS: an Illustrator for the Presentation and Visualization of Biological Sequences: Fig. 1. Bioinformatics 31 (20), 3359–3361. 10.1093/bioinformatics/btv362 26069263 PMC4595897

[B46] Mairbäurl H. Weber R. E. (2011). Oxygen Transport by Hemoglobin. Compr. Physiol. 2 (2), 1463–1489. 10.1002/cphy.c080113 23798307

[B47] Natarajan C. Inoguchi N. Weber R. E. Fago A. Moriyama H. Storz J. F. (2013). Epistasis Among Adaptive Mutations in Deer Mouse Hemoglobin. Science 340 (6138), 1324–1327. 10.1126/science.1236862 23766324 PMC4409680

[B48] Newton D. A. Rao K. M. K. Dluhy R. A. Baatz J. E. (2006). Hemoglobin Is Expressed by Alveolar Epithelial Cells. J. Biol. Chem. 281 (9), 5668–5676. 10.1074/jbc.m509314200 16407281

[B49] Nguyen L.-T. Schmidt H. A. Von Haeseler A. MinhIQ-Tree B. Q. (2015). IQ-TREE: A Fast and Effective Stochastic Algorithm for Estimating Maximum-Likelihood Phylogenies. Mol. Biol. Evol. 32 (1), 268–274. 10.1093/molbev/msu300 25371430 PMC4271533

[B50] Nishi H. Inagi R. Kato H. Tanemoto M. Kojima I. Son D. (2008). Hemoglobin Is Expressed by Mesangial Cells and Reduces Oxidant Stress. Jasn 19 (8), 1500–1508. 10.1681/asn.2007101085 18448584 PMC2488266

[B51] Olmeda B. Villén L. Cruz A. Orellana G. Perez-Gil J. (2010). Pulmonary Surfactant Layers Accelerate O2 Diffusion through the Air-Water Interface. Biochim. Biophys. Acta (Bba) - Biomembranes 1798 (6), 1281–1284. 10.1016/j.bbamem.2010.03.008 20227386

[B52] Opazo J. C. Hoffmann F. G. Storz J. F. (2008). Differential Loss of Embryonic Globin Genes during the Radiation of Placental Mammals. Proc. Natl. Acad. Sci. 105 (35), 12950–12955. 10.1073/pnas.0804392105 18755893 PMC2529089

[B53] Padró T. Van den Hoogen C. M. Emeis J. J. (1990). Distribution of Tissue-type Plasminogen Activator (Activity and Antigen) in Rat Tissues. Blood Coagul. Fibrinolysis 1 (6), 601–608. 2133239

[B54] Pérez-Gil J. (2008). Structure of Pulmonary Surfactant Membranes and Films: the Role of Proteins and Lipid-Protein Interactions. Biochim. Biophys. Acta 1778 (7-8), 1676–1695. 10.1016/j.bbamem.2008.05.003 18515069

[B55] Possmayer F. Nag K. Rodriguez K. Qanbar R. Schürch S. (2001). Surface Activity *In Vitro*: Role of Surfactant Proteins. Comp. Biochem. Physiol. Part A: Mol. Integr. Physiol. 129 (1), 209–220. 10.1016/s1095-6433(01)00317-8 11369545

[B56] Qi X. Z. Wang X. J. Zhu S. H. Rao X. F. Wei L. Wei D. B. (2008). Hypoxic Adaptation of the Hearts of Plateau Zokor (*Myospalax Baileyi*) and Plateau Pika (*Ochotona Curzoniae*). Sheng Li Xue Bao 60 (3), 348–354. 10.3321/j.issn:0371-0874.2008.03.006 18560725

[B57] Qiu Q. Zhang G. Ma T. Qian W. Wang J. Ye Z. (2012). The Yak Genome and Adaptation to Life at High Altitude. Nat. Genet. 44 (8), 946–949. 10.1038/ng.2343 22751099

[B58] Reynafarje C. Faura J. Villavicencio D. Curaca A. Reynafarje B. Oyola L. (1975). Oxygen Transport of Hemoglobin in High-Altitude Animals (Camelidae). J. Appl. Physiol. 38 (5), 806–810. 10.1152/jappl.1975.38.5.806 1126888

[B59] Sakamoto Y. Ishiguro M. Kitagawa G. (1986). Akaike Information Criterion Statistics, 81. Dordrecht, The Netherlands: D. Reidel.

[B60] Sarkar M. Pal R. Das D. Mondal D. Mohanty T. Pattanaik S. (1999). Postnatal Persistence of Foetal Haemoglobin in Yaks. Aust. Vet J 77 (3), 190. 10.1111/j.1751-0813.1999.tb11234.x 10197251

[B61] Schelshorn D. W. Schneider A. Kuschinsky W. Weber D. Krüger C. Dittgen T. (2009). Expression of Hemoglobin in Rodent Neurons. J. Cereb. Blood Flow Metab. 29 (3), 585–595. 10.1038/jcbfm.2008.152 19116637

[B62] Schürch S. Possmayer F. Cheng S. Cockshutt A. M. (1992). Pulmonary SP-A Enhances Adsorption and Appears to Induce Surface Sorting of Lipid Extract Surfactant. Am. J. Physiol. 263 (2), L210–L218. 10.1152/ajplung.1992.263.2.L210 1514646

[B63] Semenza G. L. Nejfelt M. K. Chi S. M. Antonarakis S. E. (1991). Hypoxia-inducible Nuclear Factors Bind to an Enhancer Element Located 3' to the Human Erythropoietin Gene. Proc. Natl. Acad. Sci. 88 (13), 5680–5684. 10.1073/pnas.88.13.5680 2062846 PMC51941

[B64] Serrano A. G. Pérez-Gil J. (2006). Protein-lipid Interactions and Surface Activity in the Pulmonary Surfactant System. Chem. Phys. Lipids 141 (1-2), 105–118. 10.1016/j.chemphyslip.2006.02.017 16600200

[B65] Shams I. Avivi A. Nevo E. (2005). Oxygen and Carbon Dioxide Fluctuations in Burrows of Subterranean Blind Mole Rats Indicate Tolerance to Hypoxic-Hypercapnic Stresses. Comp. Biochem. Physiol. Part A: Mol. Integr. Physiol. 142 (3), 376–382. 10.1016/j.cbpa.2005.09.003 16223592

[B66] Shao Y. Li J.-X. Ge R.-L. Zhong L. Irwin D. M. Murphy R. W. (2015). Genetic Adaptations of the Plateau Zokor in High-Elevation Burrows. Sci. Rep. 5 (1), 17262. 10.1038/srep17262 26602147 PMC4658562

[B67] Shehee W. R. Loeb D. D. Adey N. B. Burton F. H. Casavant N. C. Cole P. (1989). Nucleotide Sequence of the BALB/c Mouse β-globin Complex. J. Mol. Biol. 205 (1), 41–62. 10.1016/0022-2836(89)90363-x 2926808

[B68] Shorr R. G. Nho K. Cho M. O. P. Lee C. Czuba B. Shankar H. (1993). Process for Hemoglobin Extraction and purificationU.S. Patent No. 5. Washington, DC: U.S. Patent and Trademark Office.

[B69] Solovyev V. V. Shahmuradov I. A. Salamov A. A. (2010). Identification of Promoter Regions and Regulatory Sites. Comput. Biol. transcription Factor binding, 57–83. 10.1007/978-1-60761-854-6_5 20827586

[B70] Storz J. F. Moriyama H. (2008). Mechanisms of Hemoglobin Adaptation to High Altitude Hypoxia. High Alt. Med. Biol. 9 (2), 148–157. 10.1089/ham.2007.1079 18578646 PMC3140315

[B71] Storz J. F. Natarajan C. Cheviron Z. A. Hoffmann F. G. Kelly J. K. (2012). Altitudinal Variation at Duplicated β-Globin Genes in Deer Mice: Effects of Selection, Recombination, and Gene Conversion. Genetics 190 (1), 203–216. 10.1534/genetics.111.134494 22042573 PMC3249357

[B72] Straub A. C. Lohman A. W. Billaud M. Johnstone S. R. Dwyer S. T. Lee M. Y. (2012). Endothelial Cell Expression of Haemoglobin α Regulates Nitric Oxide Signalling. Nature 491 (7424), 473–477. 10.1038/nature11626 23123858 PMC3531883

[B73] Tatusova T. A. Madden T. L. (1999). BLAST 2 Sequences, a New Tool for Comparing Protein and Nucleotide Sequences. FEMS Microbiol. Lett. 174 (2), 247–250. 10.1111/j.1574-6968.1999.tb13575.x 10339815

[B74] Wang X. J. Wei D. B. Wei L. Qi X. Z. Zhu S. H. Rao X. F. (2008). Characteristics of Pulmonary Acinus Structure in the Plateau Zokor (*Myospalax Baileyi*) and Plateau Pika (*Ochotona Curzoniae*). Acta Zoologica Sinical 54 (3), 531–539. 10.3760/cma.j.issn.1009-6906.2010.05.009

[B75] Warburton D. Bellusci S. (2004). The Molecular Genetics of Lung Morphogenesis and Injury Repair. Paediatric Respir. Rev. 5, S283–S287. 10.1016/s1526-0542(04)90052-8 14980285

[B76] Weaver T. E. Conkright J. J. (2001). Function of Surfactant Proteins B and C. Annu. Rev. Physiol. 63 (1), 555–578. 10.1146/annurev.physiol.63.1.555 11181967

[B77] Weaver T. E. Na C.-L. Stahlman M. (2002). Biogenesis of Lamellar Bodies, Lysosome-Related Organelles Involved in Storage and Secretion of Pulmonary Surfactant. Semin. Cel Develop. Biol. 13 (4), 263–270. 10.1016/s1084952102000551 12243725

[B78] Weber R. E. (1981). Cationic Control of O2 Affinity in Lugworm Erythrocruorin. Nature 292 (5821), 386–387. 10.1038/292386a0

[B79] Weber R. E. Lalthantluanga R. Braunitzer G. (1988). Functional Characterization of Fetal and Adult Yak Hemoglobins: an Oxygen Binding cascade and its Molecular Basis. Arch. Biochem. Biophys. 263 (1), 199–203. 10.1016/0003-9861(88)90628-5 3369864

[B80] Weber R. E. (1992). Use of Ionic and Zwitterionic (Tris/BisTris and HEPES) Buffers in Studies on Hemoglobin Function. J. Appl. Physiol. 72 (4), 1611–1615. 10.1152/jappl.1992.72.4.1611 1592755

[B81] Wei D.-B. Wei L. Zhang J.-M. Yu H.-Y. (2006). Blood-gas Properties of Plateau Zokor (*Myospalax Baileyi*). Comp. Biochem. Physiol. Part A: Mol. Integr. Physiol. 145 (3), 372–375. 10.1016/j.cbpa.2006.07.011 16945563

[B82] Widmer H. R. Hoppeler H. Nevo E. Taylor C. R. Weibel E. R. (1997). Working Underground: Respiratory Adaptations in the Blind Mole Rat. Proc. Natl. Acad. Sci. 94 (5), 2062–2067. 10.1073/pnas.94.5.2062 9050905 PMC20043

[B83] Wildman D. E. Uddin M. Opazo J. C. Liu G. Lefort V. Guindon S. (2007). Genomics, Biogeography, and the Diversification of Placental Mammals. Proc. Natl. Acad. Sci. 104 (36), 14395–14400. 10.1073/pnas.0704342104 17728403 PMC1958817

[B84] Wilkins M. R. Lindskog I. Gasteiger E. Bairoch A. Sanchez J. C. Hochstrasser D. F. (1997). Detailed Peptide Characterization Using PEPTIDEMASS-Aa World-Wide-Web-Accessible Tool. Electrophoresis 18 (3‐4), 403–408. 10.1002/elps.1150180314 9150918

[B85] Williams M. C. (2003). Alveolar Type I Cells: Molecular Phenotype and Development. Annu. Rev. Physiol. 65 (1), 669–695. 10.1146/annurev.physiol.65.092101.142446 12428023

[B86] Wride M. A. Mansergh F. C. Adams S. Everitt R. Minnema S. E. Rancourt D. E. (2003). Expression Profiling and Gene Discovery in the Mouse Lens. Mol. Vis. 9 (50), 360–396. 10.1016/S0022-2836(03)00794-0 12942050

[B87] Xu Z. Wang H. (2007). LTR_FINDER: an Efficient Tool for the Prediction of Full-Length LTR Retrotransposons. Nucleic Acids Res. 35 (2), W265–W268. 10.1093/nar/gkm286 17485477 PMC1933203

[B88] Zeng J. X. Wang Z. W. Shi Z. X. (1984). Metabolic Characteristics and Some Physiological Parameters of the Mole Rat (*Myospalax Baileyi*) in an alpine Area. Acta Biol. Plat Sinica 3, 163–171.

[B89] Zhang D. Fengquan L. Jianmin B. (2000). Eco-environmental Effects of the Qinghai-Tibet Plateau Uplift during the Quaternary in China. Environ. Geology. 39 (12), 1352–1358. 10.1007/s002540000174

[B90] Zheng S. H. Zhang Z. Q. Cui N. (2004). On Some Species of Prosiphaneus (Siphneidae, Rodentia) and the Orgin of Siphneidae. Vertebrata Palasiatica 42 (4), 297–315. 10.1007/s11670-004-0048-0

[B91] Zuo Y. Y. Veldhuizen R. A. W. Neumann A. W. Petersen N. O. Possmayer F. (2008). Current Perspectives in Pulmonary Surfactant - Inhibition, Enhancement and Evaluation. Biochim. Biophys. Acta (Bba) - Biomembranes 1778 (10), 1947–1977. 10.1016/j.bbamem.2008.03.021 18433715

