# Supplementary material for: A New Homotetramer Hemoglobin in the Pulmonary Surfactant of Plateau Zokors (Myospalax Baileyi)
Source: Front Genet. 2022 Mar 15;13:824049. doi: 10.3389/fgene.2022.824049 (PMC8967358; doi:10.3389/fgene.2022.824049)
Supplement: Supplementary file 1 [file DataSheet1.DOCX]

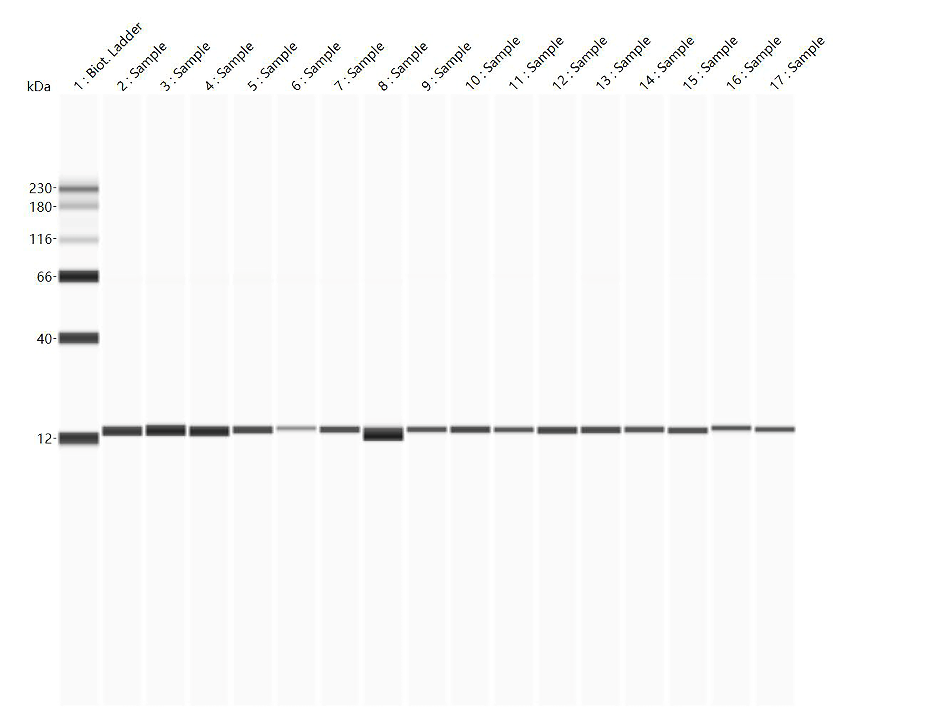


**Figure S1. The entire lane with** **molecular weight marker at the high altitude (3,700 m) and the low altitude (2, 260 m) in lung tissues of plateau zokor**. The lane 1 was the molecular weight marker, the lanes from 2 to 9 were the altitude of 3, 700 m (*n*=8), the lanes from 10 to 17 were the altitude of 2, 260 m (*n*=8).


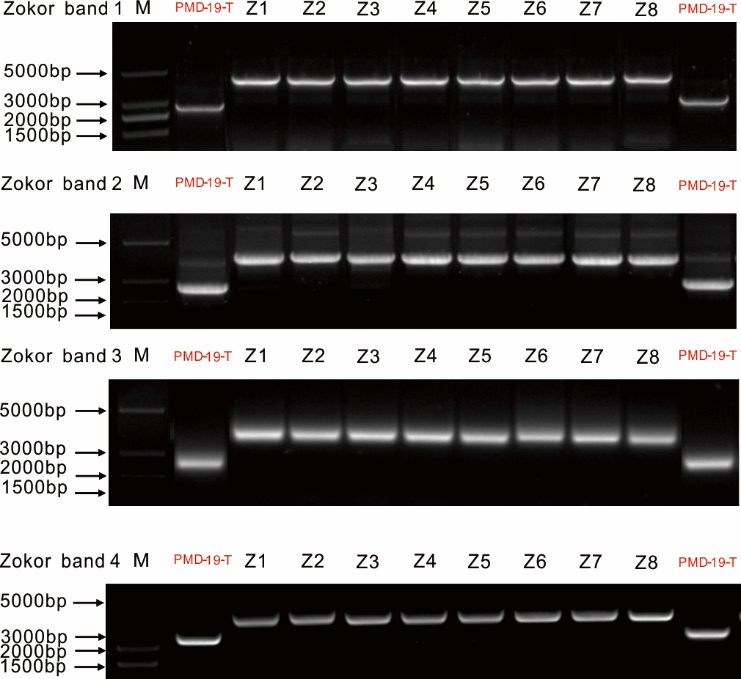


**Figure S2. PCR results of bacterial broth with *β*-like globin genes of plateau zokor.** M represented Marker, Z represented plateau zokor (*n*=8). The PCR products were ligated into pMD®19-T vector and the transformation of JM109 high-efficiency competent Escherichia coli cells were performed using the PCRR II TOPOR Vector Kit (Invitrogen).


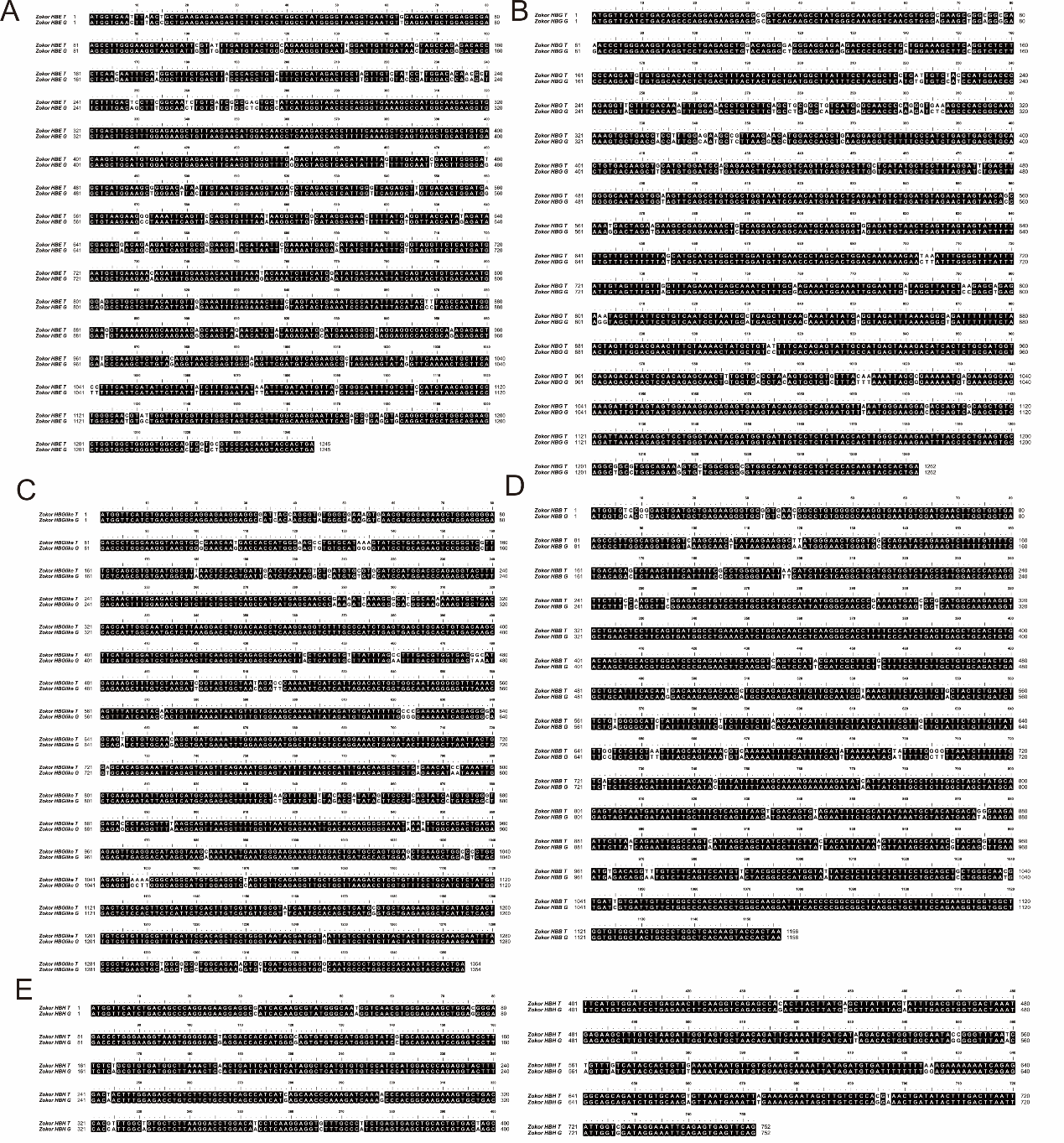


**Figure S3. The aliments between the *β*-globin genes of DNA sequencing and *β*-globin gene cluster annotating of plateau zokor. a**, **b**, **c**, **d** and **e**, The aliments of *ε*-globin gene (*HBE*), *γ*-globin gene (*HBG*), *γ*-like globin gene (*HBGlike*), *β*-globin gene (*HBB*), and *η*-globin gene (*HBH*), respectively. The suffix T and G of each globin gene name was represented the *β*-globin gene sequences got of DNA sequencing and β-globin gene cluster annotating, respectively.


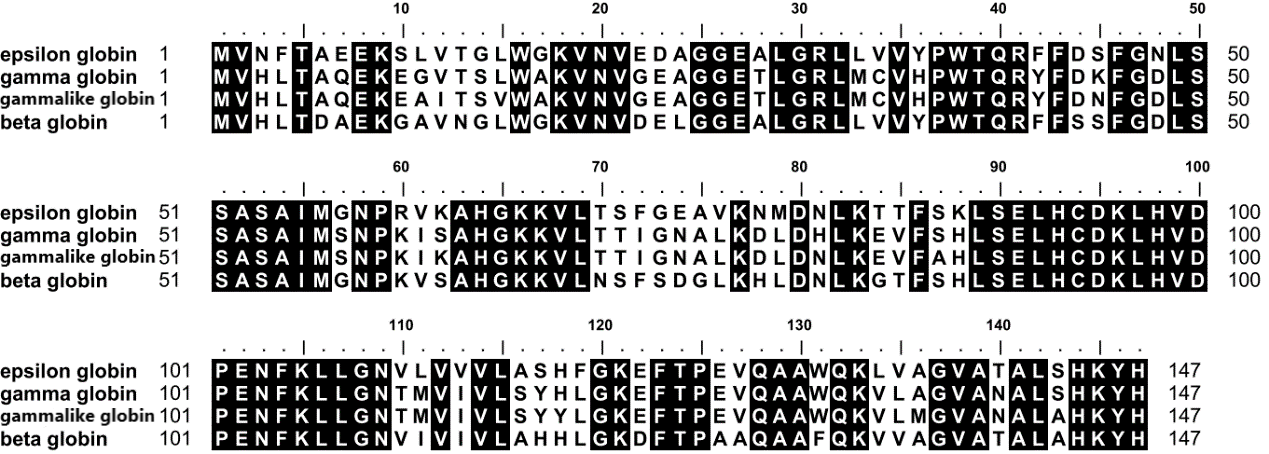


A

B


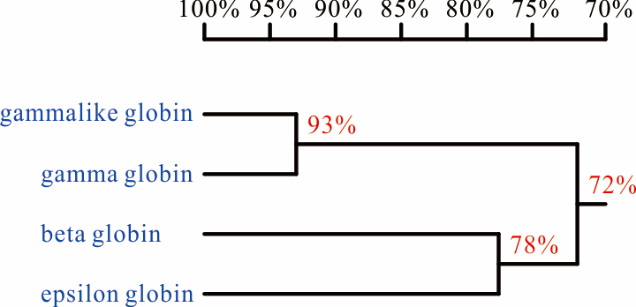


**Figure S4. The amino acids aliments among the β-like globins of plateau zokor**. **A**, the amino acid sequences of the β-like globins. **B**, the homology tree based on the β-globins reconstructed by DNAMAN 7.0.


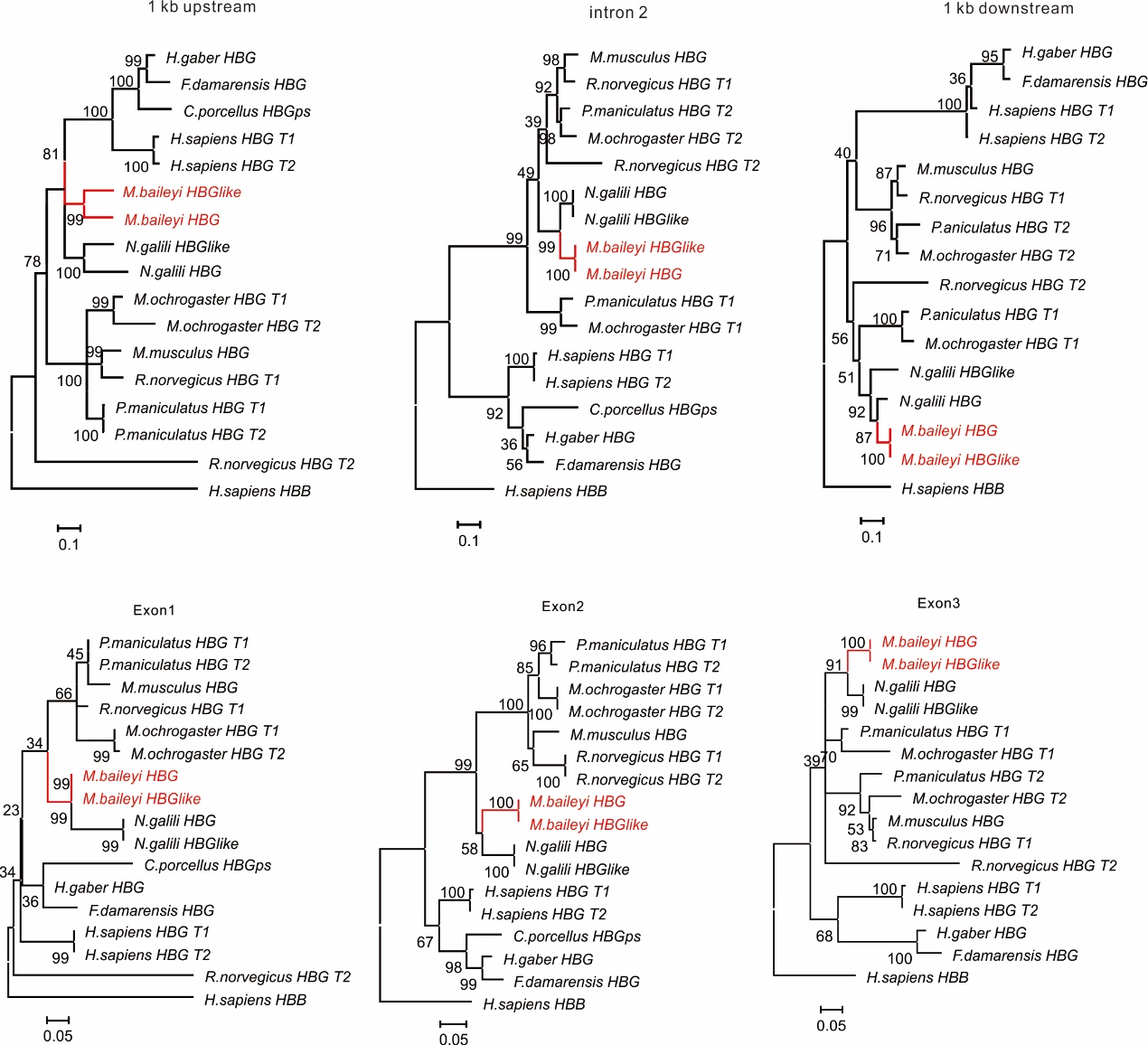


**Figure S5.** **The** **origin and evolution of the plateau zokors**′ **γ-like gene.** Bayesian inference phylograms depicting relationships among *γ*-like (*HBGlike*), and *γ* (*HBG*) gene in 11 studied species based on 1 kb of the 5′ flanking sequence (Left), intron 2 (Center), 1 kb of 3′ flanking sequence (Right) and three exons.

**>Zokor *HBGlike***

AAAACTTGAAGATAAATATTTAATTAAATGCACAGACAATTGTTGCCTACGCCTGTAATATTATTATCAATTTAGTCCATAGAGCCACAATATCAAGCCAAAGCTTCTGGCATCGTGATCTTGGATTGCTATATTTAGGGGAAAAAAGTGTCTATGTGTGAGGAAG**ACGTGC**GATACAGAC**CAGGT**TGTTCATGGGAAATTGCATCCCAGCAGAGACAGCGGGTATGAACTCTTATCTAATGGACATAACCCAGGATACTTTAGAGTGGTTTCACAAGCAAAGGAATAAGAAAAGTAGGTACTTAACAGATAATGTCTGAAATCATTAGAACAAGCAATCATTTTCATCTCCAGAGACTATCCATGCCAAGTAACATTGACACCATTTCATGAGGAAATTTAAGGCCATATTCCACTGTTGCATCATCATCCAGACCGCTTTATACTGTGGAAATAGGCATTGTCTGGTGACTGCCCAAGTATATCCACAGGGAGGCAAATACTATTGCTGAAAGTAGTCATGCTGTACTTAGAAGCATGAGAGAGGTAAGGCAGCTGACCCATAACACATCATAGCAATCTGTACCCATAAGAAACTGAGGCTGCATGGAAACTGCTTTCACTCTACCACTGTGCTCCTGGGAGACCATGGTCTAAACACTCAGGGAATGTACTGAGTAATGGCATACAGTGGTCTCTTGTAAGCTAATTCAGAGTAAGAAT**TATATATATATATATATATATATATA**TCACATATTTACTTGCATCATTCTTTAACTTTTAAAATTTTTTAAAAATATATTAAATAATTTTAAAAAAA**TCTTTGAGAATCAGAAATCTTTTTGGATCATATTAACCCCACCTCATCTCCTTCCACATCTGCCCACACCTCCCTAACCACCCAACTTACTGTTCTCTTTTTCGTTTTGTAAAGCCCCTCATATCCAATGTGTGTTGCACAAATACTCTTGGGTGTGGGACTTTCCCCTGAGCAGAGTCCACCTACCACTGGTCACACCCTTAAACAAACTGACTCTTCCTCACCCAGCAGCTATCAATTGCTAATAACTCCTCAGCTAGGGGTGGGACTCTACACTCACCTCCCCCTTCTCTGCTGGTATTTTGTCTGGTGGAAGTGTCTGCAGGTCTTCTGCTTGCTGTCACAACTGCTGGGAGTTCATATGCACAACAGGATGTTGTGTGCAGGAAACAGTGTTTCCTTTAGTCATCCACTGTTTTGGGCTCTTCCAATCTTTTTGCCCCCTGTCCACTAAATTGCCATGAACCCTGGGTAGACAGTATGAGAATATATATATATATATATAAAACTGCTGAACACTCCCTAGTCAGTTAACCTCTGCAAATTCACAAGTTGTGGGTCTCTATATTAACCACTACCTAATGTAAAAGGAAGTTT**GAGAATTTCATCCATTAACACACTATTATTTTTTCACTGAGCTCTATCTGCCCTCCAACTTCTCCCTTGTTCCTCCCCACTTCCCCTCAAATTCCTGTTCATAAAAGTAATACTTGTCTCATCTTTGACCCTGATTCTTGTGCCTAAAATAACTCTCATTATTATTTCAGATAAGTTCATTAGTCTACGCTTCAGCTGTAATCTACTGGACTTTGTAAAGTTATAATAAAAAATTTAAGAAAGATTGATAAAGAAATCCTAAAAAATCTCTTACTAAGAAGGGAAATAATCAGGTTGAAGTGAAAAATGAAAGAGAGAAGAGGAAGGCTCTAAACAGTTTAACAGTAGGGTCTTCATGAGGGGCCAACCTGACATTATCTTAATGGGAATGCTATCTAAAACTTCCTCCACTGGACCCCACCCTGGCTTGCCCAGCATCCCTTAACCAATAGACTCAGAGTACTGGCTGGCAGGGAAGGGCTAGGAGGTCCCCTGGAAAGAATAAAAGGACATGCCTTCCAGCAGCTGAACAGACTTGTTTCTAAAGAGCGGAGGTTATTGCCAAGCTCCTAGGTCAGCAACC

**TSS**

**TATA box**

**CAT box**

**TFBS**

**TFBS**

**TFBS**

**TFBS**

**NFkB (Enhancer)**

**Line/L1**

**SP**

**HBS**

**HAS**

**HRE**

**ATGGTTCATCTGACAGCCCAGGAGAAGGAGGCCATCACAAGCGTATGGGCAAAGGTCAACGTGGGAGAAGCTGGAGGGGAGACCCTGGGAAG**

**Exon1**

GTAAGTGGGGAACAAGGACCACCATGGGGAGTGTGTGCATGGGGTATCCTGCAGAAGTCCGGGTCCTTTCTCAGCGTGTGATGGCTTAAACTCCACTGATTCATCTCATAG

**GCTCATGTGTGTCCATCCATGGACCCAGAGGTACTTTGACAACTTTGGAGACCTGTCTTCTGCCTCAGCCATCATGAGCAACCCAAAGATCAAAGCCCACGGCAAGAAAGTGCTGACCACCATTGGCAATGCTCTTAAGGACCTGGACAACCTCAAGGAGGTCTTTGCCCATCTGAGTGAGCTGCACTGTGACAAGCTTCATGTGGATCCTGAGAACTTCAAG**

**Exon2**

GTCAGAGCCAGACTTACTCATGTGCTTATTTAGAATTTGACGTGGTGACTAAATGAGAAGCTTTGTCTAAGATTGGTAGTGCTAACAGATTCAAAATTCATCATTAGACACTGGTGGCAATAGGGGGTTTAAACAGTTTATCATACCACTGTTTAAAATAATGTTGTGGAAGCAAAATTATAGATGTGATTTTTGGGGGAAAAATCAGAGGGCAGCAGATCTGTGCAAGAGCTGATGAAATTTGGAAGGAATGGCTTGTCTCCAGGAAACTGAGATACTTTGACTTAATTACTGGTGCACAGGAAATTCAGAGTGAGTTCAGAAATGGAGTATTTCTAACCCATTTGACAAGCCTCTGAGAACATAATAAATTGCTCAAGAATATTAGGTCATGCAGAGACTT**TTTCCTCTGTTTGTTCT**AGACCTTATACTTCCCTGAGTATCCTGTGTGGCTGAGAGCCTAGGTTTAAAGCAGTTAAGCTTTTGGTTAATGAGAAATTGACAAGAGGGGGAAATAAAATTGGCAGACTGAGAAGAGTTGAGGACATAGGTAAGAAAATATTGAATGGGAAGAAAGGAGGACTGATGCCAGTGGAACTGAAGCTGGACTCTGCAGAGGTGCTTGGGCAGGCATGTGGAGGTCCAGTGTTCAGAGGTTGCTGGTTAAGACCTCGTGTTTCCTGCATCT**CTCATTCTCACTTGTCGTGTTGCGTTTCATTCCACAGCTCATTCTCACTTGTCGTGTTGCGTTTCATTCCACAGCTCATTCTCACTTGTCGTGTTGCGTTTCATTCCACAG**

**SP**

**ETS/NFAT**

**CTCCTGGGTAATACGATGGTGATTGTCCTCTCTTACTACTTGGGCAAAGAATTTACCCCTGAAGTGCAGGCTGCCTGGCAGAAGGTGTTGATGGGGGTGGCCAATGCCCTGGCCCACAAGTACCACTGA**

**Exon3**

GCCCGGCACCCAGATGACCACCACTCCTCTGTGTGCTTTACTTCCTTCTTCACATGAGCACTTGACTGGACCTTGAGTTCACAGCTTCTGATC**AATAAAGA**TCATTCTTTTCAGCAATGGAACTTTGATGTTTTGTGTGGTCTGTTTTTCTCTGTTGTGTTTATGTTGTTTTATATTCTGGGACTACTGGGATGGAATTTTGTGACTTGAATAAAGCTTACTCAAAGAGGGAGCACATCATCATTGTTACAATAGGAAATGACCAGAGGATATAGTATGAACAGTAAGTTCAGTCTACGCTCTTGCAGGTAGCACTTCA**GAGGGAAGGA**AGATTGCAAGTTCAAGGCTCACCCAGGCAACTGAGTGAATCCTAGGTTAAACAGTATAAATTAATGGCACTTGGTGTCATAATGAAATAAGCAAAATAGGATGGTTGCCAGGAGATGAAAGCCCTATTTTTATCCCCAGTAACACT**ATACATACATATACATGGGTATGTATATATATATTCACAATGACTGTATATATACACATATATATGTATATATATGTATGCA**ATGACTGAAACAATCAGTATCATTACTGTATTATCCAAGATAGAGCATTATTAAAAATAATCTCTTCATGGTTATAAAATTGATTCAAATTTTCATAAATCTGACATCAGTTTTAAGATTTAAATCTAAGAATTGAGACATATAAATAAGGAATGAACAAAGGTATAATGACAAAGTCCTGGTCTCAAATTTGCCGGTGTAAGAGGAATTGTAACAAGGCAAAGGATGGCAGAAATTGCCCAGCATGTGTGAATATATCCAGGAGTTAACCTGGTACCCTCATTGACTCACATCTATTTGAGAGTGCAGAGTTGCTTTGGGAGGCTTTTTTTGAGATTTTTAAAAATCAGAAAGTAGAAAGCTATATCTTAGAAAACATACCTGCCCATGATCACTTCCTAGACTCCATGTGTTGGTCAGATTTTGACTGGTTAACTATATTAAAATTTGTCTCTTTATTTTCCCTAAACATGGCAATCTTAAATTTGAAGCTTTGTCTTGGTTTTATGCCTCAGTGACCTGAATTAATATTATAATGGAAAGGAGGAAAATATTTGTGCATTTAATTAAAGATTTATCTTCTAATCCTCTTCAAAGAAGGAAACCAATAATTCTTTTTGTTTAATTCCAGGGTATTTCTGAGCACTGTGACCTTAAATATAACTCAGAATTATACAACCAATATTTCCTTATTTTGCTCAAAATTAGTTTGCCAAGTTGGTGAGAACCATGTGCATTTTTAATAATAAGTGTCCATTTCTCCTCTCCATGGTCATTTTACTTAGATACCTGGATGGACAGAAGTCAGACTTTCTACCTGTTAGGAAGATATAGATATATGTATTTCAAATCATACATCCCAGACAACATAATTCAAATGTAATTCATAATTTATACTCACATTTCCATGGATGAAAAAATATTTTAATAATGAAAGGAAAATTTTTTCCATTTAACTCTCTCCATCTATGAAAATATTTGTAATTTCAAAGATCATACAAATTAAATTAATTCACCACCATTGTTATTATTATTTTTTAAATGTACTTATGCGTGTACATTAGTGAGGAGTTGATATGTATGTGTGCTTTCATGTATGCCTGTAGGTTTGGAAGCAAGAGAAACACCTTAGTTCTTTTTCAGGCGTTGTCCATCTTGTCATTTAAAAAAATTTTTTAAATTTTTTTCATTTTACACATATAATGGTTTTGTGCCTCA**GTGTATATGAGTTCCTGCATATATGTGAGTGCACTCATGTTGTGCGATGCCTGTGGAGGTCAGGAGAGGGCGTTGGATGGAGGTCAGGAGAGGGCGTTGGATCCCCTGGACCTGGATCTAAGCATGGTTGCTAGCTTCCATGTGAGTGCTGGGAGGTGACCCTGGGTCCTCTGCAAGAGCAGTCAGTCTTCTTAACGGCTGAGCCATCTCTCTGCC**ACCCATCTT

**Poly-A**

**Ets1**

**LTR/ERVK**

**ERVK (Enhancer)**

**Sine/B3**

**>Zokor *HBG***

CAGTCCAAATTCCAAGACAAATTGAAAAGTAATTCATAATTTATGAACATACTTAGTCACATGTATAGGTTTGAATGACTCAGGATTTTTAACAATACATGTTAAATAATTATTTTTGATAGTGAATATGTTAACTGAATGAAATTAGTGAAGAGTTTAGCAGTATCTCCATGG**TGTTCTGGTAATTTTTATTGTCAACTTGATATAGTCTAGAATCACCATGGAAGAAGGAACCTCGTTTGAAAAGTTTTCTTCTACCACACTGCCCTGTGGGCATGCCTTTCAGGCATTGTTTTGATTACTAATTGATGTAGGAGGGCTCAGTCCATTGTAGGAGGCACCATCCCTAGACCCATGTGCTTGGGCTGTGTAAGAAAGCTGGATGAGCGTGATCCAGAGAGAGACAGTCAGCAGCCTTCCTCCATGGCTCTTGCCTCCAAGTTCATGCTTTGAGTACTCGCTTTGACTTCCTTCAGCTATGGGTTATAACCTAGAAATAAAAGCCTGAATAAGCTTTTTCCTCACAACTTGCTTTTGATGAGTATTTTATCAAAGCAACAGAATGAAGCTAGAACA**CTTGGAATAGTATTGGAAGAGTATACAGTATTTAGAGATTATGTCATTACTAGGATTAAATCTTACAACAGCTCCTCATTTCCAACCATGGAAGATGAGTTCTATGTCACTAGGTCCATAGTTGCTCTGAAACAGTGCAACCTG**CCAAT**CCTCACATTATTATCTGTGCTCTCTTCACCAGTCCATTATGAGTTTCTGTATCATACAAGTAGTTTTCTTCAACTTCAGAAGACTGTCATTCCAACTTCTAACCTTTTTCTCCAGTAACTTCCCAGGGCAGCTCACTTCTAGTCATTCTGGATGCAACAGTTATTTCCTCCTTAGATGAGGGGAGAGTGAGAGCC**AGAGAGAGAGAGAGGGAGAGAGAAAGAGAGAGAGAGAGAAAGAGAGAGAGAGAGAGAGAGAGAGAGAGAGAGAGAGAGAGAGAGAGAGAGAGAGAG**AAGACATGTCAGGAGCATAACTGAACTACAGATGAAGTTTATAAGGTTCAGTTATGTTCCACCTGGAACACTGTGTGTGGACTATTACTCCGTGGAAATATCCCCTGATTCTTCAGAGCTATTTTGCAAACACACAAAAAGCAAAAATACAGCCT**TGGGC**ACATGGTGTGAGAGATGTC**AGGAAG**TTTCATGAGTACAGAGAAGGCTGTGCTCAAACACTGGGTC**AGGAAG**CAGATTACTTATGCAGTGGAAA**AGAGG**GTTGTCTGGTGACTGTAGAAGTATACTGATGTAGAGGAGAATAGTATTGTTTAAACAAAGTCGATACA**ATATGG**TTCAAATTATGGCAAAGA**TATAA**AGG**AGGAAG**TAAAGGG**TGGCA**AGTTGGTAATCTATTCCTAAA**CAGAAACTAAAAGA**TAATTGGTCTGTGGGGAAGCTGGGAGCTCATGGCCTAAACCCACAGAGAATGCATTCATAGATCAACATAGTACATGCCCTTGGAAATCCCTTCTCAGGTTTATATTTGTCTCACCTGAGAACCTAATTCCAATGACCCTAAAGACTCTTATTATTCCTGGATGCTTTTTTATAATTGTCTATGCTTCTAGTTTAAT**TTACTGACCTCT**TTAAGGCAAGAGACAAAAAAAAAGAGTTTAACAATGAAGCAATGAAATCCTAAATCTCCTATTGGGAGAAGGAACCAAACAAGAGGAAAGAAAGTGAGAGGACAGGAAGCTCTAAAAGTTTAACACTAGGGTCTCCGTGAGGGGCCTACATGACACTATCTTAAAAGCGAAGGCTATCTGACCCTTGCTCCACTGGACCCCACCCTGGCTTGCTCTGTCCCCT**TGACCAATAGCCT**CAGAGTATTAGGGAGGGAGAAAGGGGCTAGGAGGTCCCCGGGGAGAATAAAAGGACATGCCTTCTAGCAGCTGAACACATTTGCTTCTGAATACCTTTGGCTATCACCAAGCTGCTAGACCCGACACC

**ISRE**

**TATA box**

**TFBS**

**TFBS**

**TFBS**

**TFBS**

**TFBS**

**TFBS**

**TFBS**

**CAT box**

**SP**

**LTR/ERVL-MaLR**

**NMP-2**

**Coup-TFⅡ**

**ATGGTTCATCTGACAGCCCAGGAGAAGGAGGGCGTCACAAGCCTATGGGCAAAGGTCAACGTGGGAGAAGCTGGAGGGGAGACCCTGGGAAG**

**Exon1**

GTAGGTCCTGAGAGCTGGACAGGGGAGGGAGGAGAAGACCCCGCCTGCTGGAAAGCTTCAGGTCTCTTCCCAGGATGTGTGGCACACTCTGACTTTACTACTGCTGATGGCTTATTTCCTAG

**GCTCATGTGTGTCCATCCATGGACCCAGAGGTACTTTGACAAGTTTGGAGACCTGTCTTCTGCCTCAGCCATCATGAGCAACCCAAAGATCTCAGCCCACGGCAAGAAAGTGCTGACCACCATTGGCAATGCTCTTAAGGACCTGGACCACCTCAAGGAGGTCTTTTCCCATCTGAGTGAGCTGCACTGTGACAAGCTTCATGTGGATCCTGAGAACTTCAAG**

**Exon2**

GTCAGTTCAGGACTTGCTCATATGCTCCTTTAGGATTTGACTTGGGGCAATAGTGGAAGTTCAGCCTGTGCCTGGTAATCCAACATGGATCTCAGAATGTCTGGATGTAGAACTAGTAACAGCAAATGACTAGAAGAAGCCGAGAAAACTGTCAGGACAGGCAATGCAA**GGGGTGGAGATGTAACTCAGTTAGTAGTATTTTTTTGTTTGTTTTTTAGCATGCATGTGGCTTGGATGTTGAACCCTAGCACTGGACAAAA**AAGAATAAATTTGGGGTTTATTTATTGTAGTTTGTTGGTTTAGAAATGAGCAAATCTTTGCAAGAAATGGAAATTGGAATTGATAGGTTATCTAAGAGCAGAGAAATAGCTTATTCCTGTCAATCCTAATGGCTGAGCTTCAAGAAATATATGAGGTAGATTTAAAAGGCAGATTTTTTTCTAACTAGTTGGACGAACTTTCTAAAACTATGCTGTATTTTCACAGAGTATTGCCATGAGTAAAGACATCACTCTGCGATGGTCAGAGACACACTCCACAGAGCAACTTTGCTGCCCTAAAGTGCTGTGTTTACAAAAATTAGGCGAAAAATGAGAAAGGGAGAAAGATTGTAGTAGTGGAAAGGAGAGAGTGAAGTACAGAGGTCAGAATGTTAAATGGGAAGGAGACCAGTCGCAGCTGTCAGATTAAACACAG

**Sine/ID**

**CTCCTGGGTAATACGATGGTGATTGTCCTCTCTTACCACTTGGGCAAAGAATTTACCCCTGAAGTGCAGGCTGCCTGGCAGAAGGTGTTGGCGGGGGTGGCCAATGCCCTGTCCCACAAGTACCACTGA**

**Exon3**

GCCCTCTTTCTGGCTGACCAGTGCTCCTGTGTGTCCCCCATT**CCCTCCCTC**TACACCTGAGTACTGAACTGGGCCTTGAAAGCCCAGGTTCTGTTT**AATAAAAA**TCCTTCTCTTCAGAAATTGAAAAACAATATTTTGTATTGTCTGTTTTTACTTTTATGATAAATAAACATAAAAAGTTGATGGGTTCCTGGGAAAGGAAGGACTTAGGTAGGAAAAACTAACTTTTCTGAAAAGTCTTTAGGAATCAGGAAAATGAATGTTATGGGAGAGTACTGGAGAGCCAAGAAGACTGAAAAGATGGGGGGG**GGGGCGGGG**GAGTGTCATGGAGTGNNNNNNNNNNNNNNNNNNNNNNNNNNNNNNNNNNNNNAAAGGACTGAGAAGGAAGGAAGAAGGAAGCGTGTGGAAATGTAGCAAAAATTTACTATACGGTTGAGTAAAGAAGAGGTTGAGCAGTGGTTACCGAGAAGGAGGAAATATGGGACACCCTAAATTTCTTAGAAGAACTTTATATAAATATGTATCTAAAGTCAAAGATGGAATTATTATTTTTAATTTACTTGAATGTACATTTTGAAATAGATGGCTTCTACAGATTACTTACATATGTAATCATGGAAGACTGATCTTTTAGAATAAGGGAGAATTTGGGTGATAATGAGGAACTGTCCAGAAGAAATGCCCATGTCTCAACTGCAGATGACTGTTCCATGTGTGGGATTCGGTTTCATTGTGTCACTAGGAAGGAAAGCTATGGTGCAGTCACTGGGCTGTGTCTGCGCAATATACCCTAGATGTCCTAGAATGAACTTAGTGTGAGGATTTAAGTGTTGGCAGCAAGTGAAAATGATGAAGTTACATAGACACAAAGTCGACGTGAGTTTAAAAGGCTCATAGTCTGAAACTTGATTGGCAGCGTGATGACTTGAACATTACTCAAAGGAAAGTACAGGCAAACATATCAGGGCTTGAGATCTTTAAACATTGGCAGTTAGAGTTCTGTATGCAGAATAATAACTGTACCACTCATTTCTGAGACTCCATTTATTGGTCAGAATTTAACTGGTTAATTATATTTAAAATTGTCTCTGTCTCTGCTATAAGGTGCCAAGAAAAATCATTCTCATAGATTCAGTCTAGGGTCTTTTTGAACAATAAATCTTTCAATATGGCATAAGTAGAATAAACAACATTCATCCACTGTGCTCAAATTTATTTCACAAAGTTAATGAGAACCATGTGGAGTATTTTTTTTTTTCTCTTTCCTGCCTCCTCCTCCAATATTATTCTGTTTCTGGATACCTGGGGATGAAGGACCTTGATGATGGAGCAATGGGAAGAACATAAGACTTTCTACCTCTTAGAAAGGTAGACAGAGATCTATATAAATCTAACCAAATTCCCAAGAAAACAAACTTAAAAATATTTCATAATACACACTCACATATGGTGGTAGTTCAAGAATTTTAACAAATGAATGATGAGTAATTCTTGGCCAGTTAGCTATCTCTTTTAAATTATATAAAATGTATGAATGATGAGTAATTCTTAGCCAGTTAGCTATCTCTTTTAAACTATATAAAACGTATAAGGAACAATAAAGATTGAAATCAACACATCTCCATCATCACTGTG**ATTATTATTCATTATTATT**GTTTTATGTGTTTGTGTGCATGTGTGTAAGAAAGGAGGTATACAAGGAGGTATGCA**TGTGTGTATACATGCATGTGTGGAAACCAGAAGACAGCCTTATTAGTCATTCCCTCAGGTTCTTTCCATCTGGTTATTCTTGAAACAGGGTCTCTCACTGATCTTGAACTCAACAGATAGGCTGGGCTGGCTGGCCAGCAAGCCTCAGGGATCTGAACATCTCTGCCTTTCAGGCTCTAGGATTGCAAGCAGGCCTCATCATTGCTGGTTTTAACATTTCTTTTAA**ATAATTTTTTATTTGAAAATTTAGTATAACACATTTTAATCATATTCAAGCCTCCATTTTCTCA

**Sine/B4**

**LTR/ERVL-MaLR**

**CLE0**

**SP**

**Sp1-3**

**Poly-A**

**CT/ACCC**

**Sine/B3**

**Figure S6. The promoters, transcription factor binding sites, retrotransposons, repeat sequences and exons of *HBGlike* and *HBG* of plateau zokor.**
